# Supplementary material for: Mapping the organisational and interventional framework for patients admitted to Hospital-at-Home for acute illness in Scandinavia – a scoping review protocol
Source: PLoS One. 2025 Aug 4;20(8):e0328645. doi: 10.1371/journal.pone.0328645 (PMC12321056; doi:10.1371/journal.pone.0328645)
Supplement: S1 Table — (DOCX) [file pone.0328645.s001.docx]

S1 Table Template of data extraction sheet that will be used

| **Study and Participant Characteristics** |  |
| --- | --- |
| Variables | Descriptions/Details |
| Authors, year | Names of authors and publication year |
| Country | Country where the study was conducted |
| Locality | Where the study was conducted (urban versus rural versus semi-urban) |
| Study design | Type of study (e.g., RCT, cohort, qualitative) |
| Objectives | Main aims or research questions |
| Sampling technique |  |
| Overall sample size |  |
| Sample size of participants admitted to HaH |  |
| Any comparator population |  |
| Study population | Description of the participants |
| Comorbidities | Reported comorbid conditions of participants |
| Inclusion criteria | Criteria for participant inclusion |
| Reason for admission | Why participants were admitted to HaH |
| **Intervention and Outcomes** |  |
| Variables | Descriptions/Details |
| Interventions | Types of interventions delivered |
| Involved professionals | Healthcare professionals involved (e.g., nurses, physicians) |
| Roles of healthcare professionals | Specific roles of healthcare professionals |
| Responsibility for treatment | Who holds primary responsibility for treatment decisions |
| Time period for HaH | Is HaH a possibility 24/7 |
| Outcome measures | What outcomes were measured (e.g., mortality, readmissions) |
| Results | Key findings related to outcomes |

|  |  |
| --- | --- |
|  |  |
|  |  |
|  |  |
