# Supplementary material for: Mapping the organisational and interventional framework for patients admitted to Hospital-at-Home for acute illness in Scandinavia – a scoping review protocol
Source: PLoS One. 2025 Aug 4;20(8):e0328645. doi: 10.1371/journal.pone.0328645 (PMC12321056; doi:10.1371/journal.pone.0328645)
Supplement: Attachment — Submitted filename: PONE-D-24-27648_R2_Mickael Essouma comments.pdf [file pone.0328645.s007.pdf]

PLOS ONE

Mapping the organisational and interventional framework for patients admitted to Hospital-at-Home for acute illness – a scoping review protocol

--Manuscript Draft--

**Manuscript Number:**	PONE-D-24-27648R2
**Article Type:**	Study Protocol
**Full Title:**	Mapping the organisational and interventional framework for patients admitted to Hospital-at-Home for acute illness – a scoping review protocol
**Short Title:**	Scoping review protocol: Framework for Hospital-at-Home for acute illness
**Corresponding Author:**	Kristina Kock Hansen, Ph.D.<br>Region of Southern Denmark: Region Syddanmark<br>Aabenraa, DENMARK
**Keywords:**	Hospital-at-home; acute medical illness; organisational structures; interventions, patient characteristics
**Abstract:**	Objective<br>This proposed scoping review aims to map the components of Hospital-at-Home (HaH) organisational structures and interventions for the acute care of adults.<br>Introduction<br>Healthcare systems face increased pressure due to ageing populations having multiple morbidities, leading to strains on hospitals. Hospitalization is associated with higher risks of adverse events, particularly among older individuals. The surge in hospital admissions for older adults necessitates prioritising resources and exploring alternative solutions. HaH has emerged as a promising intervention, showing favourable outcomes in patient satisfaction, stress reduction and quality of life. However, the success of HaH depends on various factors, including organisational collaboration, intervention complexity and patient demographics.<br>Inclusion criteria<br>Studies based on adults with an acute medical illness requiring emergency care and initial contact with an emergency department (<24 hours), out of hours doctor or general practitioner, under the medical responsibility of the hospital, will be included.<br>Methods<br>We expect that this proposed scoping review will identify Scandinavian HaH solutions that have been part of a research process. Peer-reviewed studies from the databases Medline, Embase and CENTRAL will be included. Studies focusing on acute HaH solutions delivered in patients' homes will be considered. Data extraction will include information on study and patient characteristics, organisational structures and intervention details as well as effect outcomes. This protocol has been registered with Open Science Framework (DOI: 10.17605/OSF.IO/WKE7X).
**Order of Authors:**	Kristina Kock Hansen, Ph.D.
	Maria Klitgaard Christensen
	Christian Backer Mogensen
	Peter Biesenbach
	Jette Holt
	Pia Lysdal Veje
	Mette Elkjær
	Caroline Moos
**Opposed Reviewers:**	
**Response to Reviewers:**	Response to Reviewers<br>We would be grateful if you could please revise the manuscript to respond to the comments raised by the Academic Editor and reviewers, and resubmit your manuscript files online once the necessary revisions are complete. Please accompany this resubmission by a detailed rebuttal letter that includes a point-by-point response to the

Powered by Editorial Manager® and ProduXion Manager® from Aries Systems Corporation

concerns raised by the reviewers and Academic Editor and details on the revisions carried out on the manuscript since its original submission. Please also submit a "Track Changes" version of the MS file in the File Inventory. You may submit some of the files provided in your appeal request if appropriate.

We suggest that you take particular care to justify the changes to the focus of your review since your last submission. One of the goals of Study Protocols is to increase transparency. Therefore, we would recommend an explicit statement within the manuscript detailing the reasons for changes to the methods whilst this protocol underwent review, and justifications for doing so.

Response: Thank you for considering our appeal request and the opportunity for our protocol to be reconsidered. We appreciate the opportunity to provide clarity to the issues raised. We have revised the manuscript according to the comments raised by the Academic Editor and reviewers and included a point-by-point response to the concerns raised. As suggested, we have focused specifically on a transparent explanation of the change in search strategy that narrowed references to Scandinavia. The comments from reviewers have also resulted in us specifically addressing the chosen "analytical" approach narrative synthesis.

Reviewer #1:
Dear editor and authors,
Thank you for the opportunity to review the revised version of this scoping review protocol on Hospital-at-Home (HaH). The authors have clarified their manuscript in a thorough manner with well-founded revisions based on the comments from the reviewer. One aspect has, however, been added to the revised version without being specified in the initial version of the manuscript, neither asked for by the reviewers. That is the narrow focus on the Scandinavian perspective, absent in the initial version. I see how the introduction has been revised according to the Scandinavian focus, but I was surprised of this specification of inclusion. I also see how this narrow focus might conflict with the broad focus on the time perspective of the reported research. Would the authors share their rationale for the Scandinavian specific focus?

Response: Yes, the Scandinavian perspective was added to the revised version of the protocol. Our stakeholders were only interested in a review of HaH within the Scandinavian region. Our research team discussed that studies identified from other healthcare systems were perhaps not useful as they could not be easily implemented into a Danish context. Furthermore, as the protocol was not yet published, we were of the opinion that we had flexibility to make refinements to ensure the review remained as comprehensive and relevant as possible. Therefore, this adjustment aimed to ensure that our findings could directly inform the development and implementation of acute HaH programs in a context relevant to Denmark. Denmark´s healthcare is characterized by universal access, strong integration between primary and secondary care similar to other Scandinavian countries.
We have carefully documented and justified all modifications to maintain transparency and integrity. We hope this explanation clarifies our rationale and demonstrates our commitment to maintaining methodological rigor.

Reviewer 1: I acknowledge the risk of selection bias if adding a time limit; however, the context of specialized hospital-based services has changed greatly the last some-thirty years, in addition to the specific application of HaH in these settings – with a supplement of digital homebased services or communication. I cannot see the consistence in the authors argumentation between a broad time and a narrow context, and I worry the scoping will be in-consistent and result in a less stringent scoping overview. I would be interested in the reflections of the authors in this matter.
We acknowledge that hospital-based services and the application of HaH programs have evolved significantly over the past three decades. However, we are still interested in capturing all relevant developments within Scandinavia. HaH is a relatively new area of focus in Scandinavia and we were interested in all studies regardless of their age. Narrowing the timeframe could risk excluding research describing the initial implementation of HaH and the thoughts behind it. We hope this explanation clarifies our rationale and demonstrates our commitment to producing a focused, comprehensive, and impactful review.

Powered by Editorial Manager® and ProduXion Manager® from Aries Systems Corporation

	Reviewer #2:<br>The authors have provided an insufficient revision that raises concerns. A Scandinavian focus was not clear from the initial version, raising questions of the study's rigour.<br>Response: See response above to reviewer 1<br><br>Reviewer 2: It is entirely inappropriate to not present an analytic plan in a protocol. A brief mention of a "narrative summary" is inappropriate. If the authors will not assess bias and/or quality, they cannot adequately present a synthesis of the literature.<br><br>Response:<br>Thank you for raising this important point and offering us the opportunity to explain in more depth. We will elaborate on our rationale for not including bias and quality assessments in this letter and will in our manuscript incorporate a more in-depth explanation of narrative synthesis.<br>As the primary aim of this scoping review is to map the breadth and characteristics of HaH solutions, including patient populations, interventions and organizational structures, it aligns with established guidance that scoping reviews are not intended to synthesize or critically appraise evidence as a systematic review would. Instead, they aim to provide a descriptive overview to identify knowledge gaps and understand the extent of the available literature (Arksey & O'Malley, 2005).<br>The inclusion of bias and quality assessments is not a standard requirement for scoping reviews, as noted in the JBI guidance on scoping reviews (Aromataris et al. 2024). Scoping reviews often focus on descriptive mapping rather than synthesizing results or evaluating the certainty of evidence. For example, it is appropriate to extract and descriptively map results without attempting to assess the certainty or quality of individual studies (Munn et al., 2018). This approach is particularly relevant for our review, which seeks to understand variations in HaH models and the contextual factors, rather than to draw definitive conclusions about intervention effectiveness.<br>For this review, we have opted for a narrative synthesis approach to systematically map the data while maintaining transparency and clarity in our methods. Please see line 181-185 in the manuscript. To support our methodological choice, we refer to Popay et al.'s (2006) guidance on narrative synthesis in systematic reviews, which highlights that narrative synthesis is a robust and widely accepted method, particularly when handling diverse and heterogeneous evidence bases. The approach allows for a structured and transparent synthesis of findings without requiring quality assessments to be conducted as part of the process. Moreover, our narrative synthesis will be conducted systematically, emphasizing consistent reporting of patient characteristics, intervention types and organizational models.<br>We hope this response adequately addresses your concerns and provides clarity on why our approach, without bias or quality assessments, is methodologically sound and consistent with best practices for scoping reviews. Thank you again for your insightful comments, which have allowed us to strengthen our protocol.<br><br>References<br>Arksey, H., & O'Malley, L. (2005). Scoping studies: towards a methodological framework. International Journal of Social Research Methodology, 8(1), 19–32. https://doi.org/10.1080/1364557032000119616<br>Aromataris E, Lockwood C, Porritt K, Pilla B, Jordan Z, editors. JBI Manual for Evidence Synthesis. JBI; 2024. Available from: https://synthesismanual.jbi.global. https://doi.org/10.46658/JBIMES-24-01<br>Munn, Z., Peters, M.D.J., Stern, C. et al. Systematic review or scoping review? Guidance for authors when choosing between a systematic or scoping review approach. BMC Med Res Methodol 18, 143 (2018). https://doi.org/10.1186/s12874-018-0611-x<br>Popay, Jennie et al. "Guidance on the conduct of narrative synthesis in systematic Reviews. A Product from the ESRC Methods Programme. Version 1." (2006).
**Additional Information:**	
**Question**	**Response**
**Financial Disclosure**<br><br>Enter a financial disclosure statement that	The author(s) received no specific funding for this work.

describes the sources of funding for the work included in this submission. Review the submission guidelines for detailed requirements. View published research articles from *PLOS ONE* for specific examples.<br><br>This statement is required for submission and **will appear in the published article** if the submission is accepted. Please make sure it is accurate.<br><br>**Funded studies**<br>Enter a statement with the following details:<br>• Initials of the authors who received each award<br>• Grant numbers awarded to each author<br>• The full name of each funder<br>• URL of each funder website<br>• Did the sponsors or funders play any role in the study design, data collection and analysis, decision to publish, or preparation of the manuscript?<br><br>Did you receive funding for this work?	
**Competing Interests**<br><br>Use the instructions below to enter a competing interest statement for this submission. On behalf of all authors, disclose any competing interests that could be perceived to bias this work—acknowledging all financial support and any other relevant financial or non-financial competing interests.<br><br>This statement is required for submission and **will appear in the published article** if the submission is accepted. Please make sure it is accurate and that any funding sources listed in your Funding Information later in the submission form are also declared in your Financial Disclosure statement.<br><br>View published research articles from *PLOS ONE* for specific examples.	The authors have declared that no competing interests exist.

Powered by Editorial Manager® and ProduXion Manager® from Aries Systems Corporation

**NO authors have competing interests**<br><br>Enter: *The authors have declared that no competing interests exist.*<br><br>**Authors with competing interests**<br><br>Enter competing interest details beginning with this statement:<br><br>*I have read the journal's policy and the authors of this manuscript have the following competing interests: [insert competing interests here]*<br><br>* typeset	
**Ethics Statement**<br><br>Enter an ethics statement for this submission. This statement is required if the study involved:<br><br>• Human participants<br>• Human specimens or tissue<br>• Vertebrate animals or cephalopods<br>• Vertebrate embryos or tissues<br>• Field research<br><br>Write "N/A" if the submission does not require an ethics statement.<br><br>General guidance is provided below. Consult the submission guidelines for detailed instructions. **Make sure that all information entered here is included in the Methods section of the manuscript.**	N/A

**Format for specific study types**<br><br>**Human Subject Research (involving human participants and/or tissue)**<br>• Give the name of the institutional review board or ethics committee that approved the study<br>• Include the approval number and/or a statement indicating approval of this research<br>• Indicate the form of consent obtained (written/oral) or the reason that consent was not obtained (e.g. the data were analyzed anonymously)<br><br>**Animal Research (involving vertebrate animals, embryos or tissues)**<br>• Provide the name of the Institutional Animal Care and Use Committee (IACUC) or other relevant ethics board that reviewed the study protocol, and indicate whether they approved this research or granted a formal waiver of ethical approval<br>• Include an approval number if one was obtained<br>• If the study involved *non-human primates*, add *additional details* about animal welfare and steps taken to ameliorate suffering<br>• If anesthesia, euthanasia, or any kind of animal sacrifice is part of the study, include briefly which substances and/or methods were applied<br><br>**Field Research**<br><br>Include the following details if this study involves the collection of plant, animal, or other materials from a natural setting:<br>• Field permit number<br>• Name of the institution or relevant body that granted permission	
**Data Availability**<br><br>Authors are required to make the data underlying their research findings fully available, without restriction.<br><br>If the manuscript reports pilot or preliminary results, the data underlying those results must be made available at the time of publication. A Data Availability	N/A - No results are reported

Statement describing where the data can be found is required at submission. Your answers to this question constitute the Data Availability Statement and will be published in the article, if accepted. When legal or ethical requirements prohibit public data sharing, authors must indicate how researchers may obtain access to the data. See the PLOS Data Policy and FAQ for detailed information.<br><br>**Important:** Stating 'data available on request from the author' is not sufficient. If your data are only available upon request, select 'No' for the first question and explain your situation in the text box.<br><br>For protocols without pilot or preliminary data, authors are strongly encouraged to state how they plan to share research data from their study when it is completed or published.<br><br>If your study includes pilot data, will they be made fully available without restriction?	
**Describe where the data may be found in full sentences. If you are copying our sample text, replace any instances of XXX with the appropriate details.**<br><br>• If **no pilot data are reported** in the manuscript, please enter the following: *No datasets were generated or analysed during the current study. All relevant data from this study will be made available upon study completion.*<br>• If the manuscript reports pilot data **held in a public repository**, include URLs, accession numbers or DOIs. If this information will only be available after acceptance, indicate this by ticking the box below. For example: *All XXX files are available from the XXX database (accession number(s) XXX, XXX.).*<br>• If the manuscript reports pilot data contained **within the manuscript and/or**	No datasets were generated or analysed during the current study. All relevant data from this study will be made available upon study completion.

Powered by Editorial Manager® and ProduXion Manager® from Aries Systems Corporation

**Supporting Information files**, enter the following:<br>*All relevant data are within the manuscript and its Supporting Information files.*<br>• If neither of these applies but you are able to provide **details of access elsewhere**, with or without limitations, please do so. For example:<br><br>*Data cannot be shared publicly because of [XXX]. Data are available from the XXX Institutional Data Access / Ethics Committee (contact via XXX) for researchers who meet the criteria for access to confidential data.*<br><br>*The data underlying the results presented in the study are available from (include the name of the third party and contact information or URL).*<br>• This text is appropriate if the data are owned by a third party and authors do not have permission to share the data.<br><br>For study protocols with data management or sharing plans, authors are encouraged to briefly describe in their Data Availability Statement how data they generate will be made accessible when the study is completed. For example:<br>• Deidentified research data will be made publicly available when the study is completed and published.<br><br>* typeset	
Additional data availability information:	

Powered by Editorial Manager® and ProduXion Manager® from Aries Systems Corporation

Dr. Emily Chenette
Editor-in-chief
PLOS One

Vejle, 31 July 2024

Dear Dr. Emily Chenette,

To be considered for publication in your journal *PLOS One*, we would like to submit this manuscript with the following title: *Mapping the organisational and interventional framework for patients admitted to Hospital-at-Home for acute illness – a scoping review protocol.* We wish to have the manuscript considered as a Study Protocol, and confirm that data collection has not been completed.

The manuscript aims to map the interventional and organizational frameworks of hospital at home (HaH) programs as well as describing in depth the population characteristics, focusing on acute illness management.

Given the aging population and rising hospital admissions, there is a critical need for innovative care models like HaH. Despite its growing importance, there is limited comprehensive synthesis of the existing interventional and organizational frameworks in this field. Additional, the extensive array of HaH descriptions complicates identifying specific components that influence the planning of a potential HaH program. A clear overview of the organizational structure, including roles and responsibilities of healthcare personnel, is lacking. Implementers need detailed insights into patient criteria and feasible home interventions to design safe and effective acute HaH programs.

Our protocol addresses this gap by outlining a systematic approach to identify, analyse and synthesize relevant evidence, providing valuable insights for healthcare practitioners, policymakers and researchers.

We have closely followed the PRISMA-P guidelines to ensure transparency and reproducibility. Key elements of our protocol include a clear statement of research questions, eligibility criteria for evidence sources and detailed methods for data charting and analysis.

Considering the future strain on healthcare systems and the benefits of HaH for patients, we believe our scoping review protocol will significantly contribute to healthcare delivery and management.

All co-authors have approved this submission, and we have no conflicts of interest to disclose.

Thank you for your consideration of this manuscript.

Sincerely,

Kristina Kock Hansen
PhD, MHS, RN
Research Center for Integrated Healthcare Region of Southern Denmark
Kresten Philipsens Vej 15, 6200 Aabenraa
E-mail: kristina.kock.hansen2@rsyd.dk
Phone: +45 23622575

Manuscript

Click here to access/download;Manuscript;Manuscript clean version.docx

Mapping the organisational and interventional framework for patients admitted to Hospital-at-Home for acute illness – a scoping review protocol

Authors

Kristina Kock Hansen[1,2*], Maria Klitgaard Christensen[1,3], Christian Backer Mogensen[4,5] Peter Biesenbach[6], Jette Holt[7], Pia Lysdal Veje[8], Mette Elkjær[1,5], Caroline Moos[1,9]

Affiliations

[1]Research Center for Integrated Healthcare Region of Southern Denmark, Aabenraa, Denmark

[2]Department of Medicine, University Hospital of Southern Denmark, Lillebaelt Hospital, Vejle, Denmark

[3]Department of Clinical Research, Faculty of Health Sciences, University of Southern Denmark, Odense, Denmark

[4]Department of Emergency Medicine, University Hospital of Southern Denmark, Aabenraa, Denmark

[5]Department of Regional Health Research, University of Southern Denmark, Odense, Denmark.

[6]Department of Emergency Medicine, University Hospital of Southern Denmark, Esbjerg, Denmark

[7]Infectious Disease Epidemiology & Prevention, The National Center for Infection Control (CEI), Copenhagen S, Denmark.

[8]University College South Denmark, Department for Applied Health Science, Denmark

[9]Department of Clinical Research, University Hospital of Southern Denmark, Aabenraa, Denmark

***Corresponding author**

E-mail: kristina.kock.hansen2@rsyd.dk (KKH)

Abstract

Objective

This proposed scoping review aims to map the components of Hospital-at-Home (HaH) organisational structures and interventions for the acute care of adults.

Introduction

Healthcare systems face increased pressure due to ageing populations having multiple morbidities, leading to strains on hospitals. Hospitalization is associated with higher risks of adverse events, particularly among older individuals. The surge in hospital admissions for older adults necessitates prioritising resources and exploring alternative solutions. HaH has emerged as a promising intervention, showing favourable outcomes in patient satisfaction, stress reduction and quality of life. However, the success of HaH depends on various factors, including organisational collaboration, intervention complexity and patient demographics.

Inclusion criteria

Studies based on adults with an acute illness requiring emergency care and initial contact with an emergency department (<24 hours), out of hours doctor or general practitioner, under the medical responsibility of the hospital, will be included.

Methods

We expect that this proposed scoping review will identify Scandinavian HaH solutions that have been part of a research process. Peer-reviewed studies from the databases Medline, Embase and CENTRAL will be included. Studies focusing on acute HaH solutions delivered in patients' homes will be considered. Data extraction will include information on study and patient characteristics, organisational structures and intervention details as well as effect outcomes. This protocol has been registered with Open Science Framework (DOI: 10.17605/OSF.IO/WKE7X).

Introduction

The Scandinavian healthcare systems are under pressure with the unprecedented numbers of people reaching retirement age and living longer with multiple chronic illnesses. These demographic changes are putting pressure on hospitals [1, 2]. Older people experience healthcare systems as fragmented with gaps in coordination. Additionally, challenges in retaining and recruiting healthcare professionals worsens the situation. Furthermore, there is a political agenda to reduce healthcare costs by reducing number of hospital beds [3] or length of stay [4]. Hospitalization is associated with a higher risk of nosocomial infections, inactivity, stress and delirium, particularly in older people [5-7].

Hospital admissions for older people over 75 years have surged by 65% over the past decade [8, 9], and recent trends across Europe show a year-on-year increase in the number of patients with acute medical illnesses presenting to hospitals [3]. Studies have shown that periods of emergency department (ED) crowding are associated with increased inpatient mortality, length of stay and costs for admitted patients, with a higher probability of patients leaving the ED against medical advice or without being seen [8, 9]. For these reasons, there is a strong need to prioritise hospital resources and find alternative and innovative solutions.

One promising intervention is Hospital-at-Home (HaH) [10, 11]. HaH has been shown to reduce adverse events and distress associated with hospitalization of adults and ease the burden on hospitals [12]. The literature contains numerous definitions of HaH, leading to diverse descriptions of interventions tailored to a broad range of patient populations requiring treatment for various conditions. The composition of healthcare professionals delivering HaH care differs considerably [10, 13]. HaH programs encompass various services, including end-of-life care, early discharge or admission avoidance [3, 10]. HaH, as described in the literature, is characterised by services that differ in patient selection, clinical procedures

and operational structure [3, 10]. Evidence on HaH programs within the Scandinavian context is limited, as Scandinavia has a less developed tradition of implementing HaH services. Additionally, Scandinavian healthcare system is characterised by free, publicly funded hospitals available to all citizens through taxation, making it challenging to draw direct comparisons with HaH solutions in countries like USA or the UK.

Our study's research question was driven by Danish healthcare administrators request for evidence on HaH solutions that ensure safety and effectiveness for patients and healthcare professionals. While Scandinavia has recently implemented some initiatives focusing on home-based telehealth and HaH services, there is limited published research on these efforts. Moreover, the literature appears to lack a comprehensive overview of various HaH solutions, including key aspects such as organisational structures, patient demographics and designated responsibility of care [3].

Typically, patients (18+ years) receiving HaH care report high satisfaction levels [14-17]. They value the comfort of their home setting, a heightened sense of independence and control and improvements in physical activity, mood, sleep quality and social interactions [14, 18-21]. Both adult patients and caregivers report that HaH is beneficial as it provides dignified care while preserving the quality of life [22]. Additionally, it enables family members to participate in patient care, providing more robust emotional support during the recovery process [22], but it can be physically and mentally demanding to be a participating caregiver [23]

However, the success of HaH is challenged by the collaboration between different health organisations and sectors, the complexity of interventions and uncertainty about which patients can be offered care and who is responsible for it. A review from 2020 highlighted that there is a lack of prioritisation of patient-centred care, including improvement of multidisciplinary continuity of care in the HaH services [13]. Furthermore,

adults ≥21 years old have expressed concerns about a potential decline in the quality of care and access to help if their condition worsens [22]. This was reflected in a randomized controlled trial by Levine et al., where 157 (63%) adults declined participation in a HaH intervention [24]. Chua et al. also reported that many relatives were anxious about the extra responsibility of caring for their loved ones in the context of HaH [22].

Four Cochrane reviews have focused on the effects of HaH with varying interventions and patient populations [25-28]. Admission avoidance appears to be an effective alternative to hospitalisation for adults with chronic obstructive pulmonary disease (COPD) exacerbations [25] or palliative care requirements [26]. Research has suggested that avoiding admission by providing acute care in the home is equally efficient to hospital care for older patients with acute cardiac failure [29] or oncological populations [30]. Generally, studies that aim to avoid hospital admittance reduce patient stress, shorten the length of hospital stay and lower the cost of care [12]. Studies vary in reporting how HaH affects mortality [11, 25, 31, 32]. A newly published Cochrane review focusing on factors influencing the implementation of HaH emphasized that clear guidelines to assess whom HaH is suitable for, provision of a trained workforce and consistent communication between staff, patients and caregivers were important when developing a HaH program [33].

Many HaH reviews focus on specific illnesses among patients or alternatives to admission with broader interventions and often do not elaborate on the intricacies of the organisations, interventions or patient characteristics [2, 34, 35]. The extensive array of HaH descriptions complicates identifying specific components that influence the planning of a potential HaH solution. We lack a concise overview of the organisational structure of acute HaH programs, encompassing the roles and responsibilities of various healthcare sector personnel. Implementation innovators need additional insights into the patient criteria suitable for HaH care and the specific interventions feasible within home settings to design effective, patient and work safe acute HaH programs.

Alternative solutions are imperative in anticipation of the future strain on the healthcare system. This proposed scoping review aims to map Scandinavian organisational structures, interventions and describe population characteristics as well as effect outcomes of adult patients receiving HaH care for an acute medical illness that would otherwise require acute hospital inpatient care for a limited period.

Review question

How is acute HaH care organised, which acute interventions are provided to what type of patients and what are the measured effects?

Materials and methods

The JBI Manual will guide the proposed scoping review for evidence synthesis, including developing this protocol to reduce the risk of impromptu decision-making [36, 37]. The review will be reported according to the Preferred Reporting Items for Systematic Reviews and Meta-Analysis (PRISMA-ScR) [38]. This protocol has been uploaded to Open Science Framework (DOI: 10.17605/OSF.IO/WKE7X). The search strategy will use the PCC (Population, Concept and Context) typology [39].

Search strategy

Searches will be conducted using the databases Medline (Ovid), Embase (Ovid) and CENTRAL (Cochrane). The preliminary search strategy for Embase is illustrated in Table 1. The search strategy will be peer-reviewed by a university librarian specialist specialising in medicine and health.

Table 1. **Preliminary search strategy for Embase**

#	Query
1	exp Home Care Services/
2	Exp Hospitals/
3	Exp Hospitalization/
4	2 or 3

5	exp Hospital to Home Transition/
6	1 and 4
7	5 or 6
8	(hospital* adj3 home).ti,ab,kf.
9	(home care and (medical services or health services)).ti,ab,kf.
10	(home based adj (medical care or medical services or health services or health care or healthcare or care services or care program*)).ti,ab,kf.
11	home ward.ti,ab,kf.
12	home based program*.ti,ab,kf.
13	admission avoidance.ti,ab,kf.
14	avoid* admission*.ti,ab,kf.
15	(hospital* adj2 avoid*).ti,ab,kf.
16	(hospital* or unit? or ward? or institution*).ti,ab,kf.
17	1 and 16
18	(Home-based adj2 hospital-based).ti,ab,kf.
19	Home hospitali?ation.ti,ab,kf.
20	Virtual ward.ti,ab,kf.
21	Home treatment.ti,ab,kf.
22	Hospital-based home*.ti,ab,kw.
23	Domiciliary hospitali?ation.ti,ab,kf.
24	Advanced care at home.ti,ab,kf.
25	(Substitute adj3 inpatient).ti,ab,kf.
26	"hospital at home".ti,ab,kf.
27	outpatient care.ti,ab,kf.
28	outpatient treatment*.ti,ab,kf.
29	7 or 8 or 9 or 10 or 11 or 12 or 13 or 14 or 15 or 17 or 18 or 19 or 20 or 21 or 22 or 23 or 24 or 25 or 26 or 27 or 28
30	exp Emergency Medical Services/ or exp Emergency Service, Hospital/
31	exp Emergency Treatment/
32	emergency department.ti,ab,kf.
33	Emergency care.ti,ab,kf.
34	rapid response.ti,ab,kf.
35	same day emergency care.ti,ab,kf.
36	emergency health service*.ti,ab,kf.
37	emergency treatment.ti,ab,kf.
38	emergency service*.ti,ab,kf.
39	emergency patient*.ti,ab,kf.
40	exacerbat*.ti,ab,kf.
41	(acute* adj2 illness).ti,ab,kf.
42	(acute* adj2 assessment).ti,ab,kf.
43	(acute* adj2 service*).ti,ab,kf.
44	(acute* adj2 decompensat*).ti,ab,kf.
45	acute medical.ti,ab,kf.
46	Acute disease.ti,ab,kf.
47	30 or 31 or 32 or 33 or 34 or 35 or 36 or 37 or 38 or 39 or 40 or 41 or 42 or 43 or 44 or 45 or 46
48	29 and 46
49	Exp Scandinavia/
50	(Denmark or Faroe Islands or Finland or Greenland or Iceland or Norway or Sweden or Scandinavia or Nordic countries).ti,ab,kf.
51	49 or 50
52	48 and 51

Searching for studies from start of database to search date

The search will be developed with three facets – acute care, Hospital-at-Home and Scandinavia. Each facet will use standardised keywords from the database's controlled vocabulary thesaurus and free text words with the Boolean operator "OR". The three facets will be combined using the Boolean operator "AND". The Hospital-at-Home facet was inspired by Cochrane reviews that developed search strings for this concept, albeit in two different reviews [16, 27]. No restrictions will be applied to the search date to ensure the identification of all relevant literature [40]. Publication type will be restricted to peer-reviewed studies to ensure research of reasonable quality but is not restricted to studies based on design. We will consider experimental, quasi-experimental, observational and descriptive studies for inclusion, as well as qualitative studies that address our review questions. Forward citation searches of included studies will be performed using the Web of Science database to identify any additional literature. Grey literature searches will consist of searching in *clinicaltrial.gov* and searches of the official Scandinavian health department websites.

Inclusion and exclusion criteria

Definitions and inclusion/exclusion criteria of primary studies for this scoping review have been summarised in Table 2.

Table 2 Definitions, inclusion and exclusion criteria based on the PCC typology

	Definitions	Inclusion	Exclusion
Population	Emergency department patients<br>Definition: Patients with the onset of an acute medical illness requiring emergency care [3]	• Adults 18+ years<br>• Initial medical contact with an emergency department (<24 hours), out of hours doctor or a general practitioner	• Adults requiring care described as post-surgery, long-term or end-of-life
Concept	HaH<br>Definition: Treatment at home under the medical responsibility of a hospital with a service that manages acute illness at a level of severity or complexity that would require hospital admission if the intervention was unavailable [3].	• Interventions describing admission avoidance to hospital	• Care not provided at the patient´s residence<br>• Interventions described as post-discharge, early-discharge or self-care by the patient<br>• Individuals with only a single contact with healthcare providers, for example, mobile acute care affiliated with an ED, who do not require admission.

Context	Scandinavia	• Studies that describe HaH in Denmark, the Faroe Islands, Finland, Greenland, Iceland, Norway, Sweden, Scandinavia, Nordic countries	• Studies from countries outside Scandinavia

Study selection

After searching, all identified citations will be imported into Covidence [41]. Covidence is a web-based collaboration software platform that streamlines the production of systematic and other literature reviews. In Covidence, duplicates will be eliminated, and results will be screened independently for eligibility by two researchers in a two-step process – title/abstract screening and full-text screening. Studies not qualifying for inclusion at full-text screening will be excluded with justification. A third reviewer will be used to adjudicate any disagreements. The search findings and the study inclusion process will be illustrated in a flow diagram following the PRISMA extension for Scoping Review guidelines [42].

Data extraction

The extracted data will include the following information: author, year of publication, country, the aim of the study, research design, participant characteristics (sex, age, comorbidities), type of acute conditions, patients´ underlying illness, availability of social support, intervention description, the organisation responsible for delivering the service, the person responsible for coordinating the services, telehealth tools such as remote monitoring and the designated healthcare provider with patient responsibility. Although the focus of the review is organisational structures and interventions, study outcomes such as mortality, readmission, length of treatment, depression, anxiety, functional status, quality of life, patient and caregiver satisfaction, caregiver stress, cognitive status, morbidity or economic costs will also be extracted and presented in the results section.

Proposed data synthesis

Data will be presented in tabular form (Table 3) and a narrative synthesis [43] will be conducted, including: 1) systematic categorization of patient characteristics, organisational structures and interventions, 2) mapping of the HaH organisations, including key stakeholders and healthcare professionals with the treatment responsibility and 3) exploring similarities and differences between the HaH organisation models, to ensure replicability and clarity.

Table 3 Characteristics of included studies for HaH in Scandinavia

Source	Design and method	Reason for HaH admission	HaH patient characteristics *	Patient comorbidities (if any)	What is the intervention/ treatment/ HaH service in the home?	Who is involved in the inter-vention (the organisational structure)?	Who has the medical treatment responsibility?	Outcomes measured and results

*number of patients, mean age, % sex

Ethical consideration

Ethical approval was not required because the study does not include participants. The study is in accordance with the Declaration of Helsinki [44] and Ethical Guidelines for Nursing Research in the Nordic Countries [45].

Discussion

Healthcare systems face additional strain from an aging population living longer with many chronic conditions, exacerbating pressures on EDs, which are associated with adverse outcomes such as increased mortality and more extended hospital stays [1, 2, 8, 9]. Generally, there is a growing imperative to allocate resources efficiently while exploring innovative, sustainable and evidence-based solutions that ensure patient safety. HaH has demonstrated favourable outcomes regarding patient satisfaction, stress reduction, prevention of hospital-acquired infections and reduced healthcare costs [10, 11].

If HaH offers the same treatment as in-hospital care, it will require significant equipment, technical devices and systems to monitor readmissions and record adverse events. It is essential to consider whether there can be similar access to services and diagnostic tools to what patients receive in the hospital [46]. Some literature describes the organisational structures of HaH programs as collaborations between hospitals and municipalities or specialised acute care teams [10, 47]. Challenges arise when multiple sectors need to collaborate on patient care and share resources [48]. Additionally, patients and healthcare professionals need to feel safe and confident about the treatment received in the home [13].

By limiting the literature to Scandinavian studies of admission avoidance, there is a risk of excluding important research and experiences from other countries or interventions, which may contribute to different perspectives or solutions. However, these limits ensure that HaH solutions are more homogeneous. The narrow grey literature search may have excluded relevant internal reports, white papers, conference papers, local initiatives or innovative pilot projects. However, these limitations were included to ensure we assessed the degree of research and the evidence base for Scandinavian HaH interventions.

We expect to present an overview of the components of Scandinavian HaH solutions. This review will benefit other health planners and researchers considering developing HaH solutions by focusing on examples of organisational structures and components of interventions in acute HaH care.

Author contributions

Conceptualization: KKH, CM, ME

Data curation: CM, KKH

Methodology: CM, KKH

Project administration: KKH

Validation: CM, KKH, MKC

Writing – original draft: KKH, CM

Writing – review & editing: KKH, CM, MKC, ME, CBM, PLV, JH, PB

Guarantor of the review protocol: Kristina Kock Hansen

References

1. Cho SJ, Stout-Delgado HW. Aging and Lung Disease. Annu Rev Physiol. 2020;82:433-59.
2. World Health Organsiation. Ageing 2018 [Available from: https://www.who.int/news-room/facts-in-pictures/detail/ageing.
3. Knight T, Lasserson D. Hospital at home for acute medical illness: The 21st century acute medical unit for a changing population. J Intern Med. 2022;291(4):438-57.
4. Stevenson A, Deeny S, Friebel R, Gardner T, Thorlby R. emergency hospital admissions in England: which may be avoidable and how? . The Health Foundation. 2018.
5. Klevens RM, Edwards JR, Richards CL, Jr., Horan TC, Gaynes RP, Pollock DA, et al. Estimating health care-associated infections and deaths in U.S. hospitals, 2002. Public Health Rep. 2007;122(2):160-6.
6. Vasilevskis EE, Han JH, Hughes CG, Ely EW. Epidemiology and risk factors for delirium across hospital settings. Best Pract Res Clin Anaesthesiol. 2012;26(3):277-87.
7. Brown CJ, Redden DT, Flood KL, Allman RM. The underrecognized epidemic of low mobility during hospitalization of older adults. J Am Geriatr Soc. 2009;57(9):1660-5.
8. Bernstein SL, Aronsky D, Duseja R, Epstein S, Handel D, Hwang U, et al. The effect of emergency department crowding on clinically oriented outcomes. Acad Emerg Med. 2009;16(1):1-10.
9. Sun BC, Hsia RY, Weiss RE, Zingmond D, Liang LJ, Han W, et al. Effect of emergency department crowding on outcomes of admitted patients. Ann Emerg Med. 2013;61(6):605-11.e6.
10. Varney J, Weiland TJ, Jelinek G. Efficacy of hospital in the home services providing care for patients admitted from emergency departments: an integrative review. Int J Evid Based Healthc. 2014;12(2):128-41.
11. Arsenault-Lapierre G, Henein M, Gaid D, Le Berre M, Gore G, Vedel I. Hospital-at-Home Interventions vs In-Hospital Stay for Patients With Chronic Disease Who Present to the Emergency Department: A Systematic Review and Meta-analysis. JAMA Netw Open. 2021;4(6):e2111568.
12. Wong SY, Teo YN, Wo YJ, Lim CW, Lai YF. Distilling lessons from home hospital implementation: a narrative scoping review. Singapore Med J. 2024.
13. Wang X, Stewart C, Lee G. Patients' and caregivers' perceptions of the quality of hospital-at-home service: A scoping review. J Clin Nurs. 2024;33(3):817-38.
14. Levine DM, Ouchi K, Blanchfield B, Saenz A, Burke K, Paz M, et al. Hospital-Level Care at Home for Acutely Ill Adults: A Randomized Controlled Trial. Ann Intern Med. 2020;172(2):77-85.
15. Levine DM, Desai MP, Ross J, Como N, Anne Gill E. Rural Perceptions of Acute Care at Home: A Qualitative Analysis. J Rural Health. 2021;37(2):353-61.
16. Shepperd S, Iliffe S, Doll HA, Clarke MJ, Kalra L, Wilson AD, et al. Admission avoidance hospital at home. Cochrane Database Syst Rev. 2016;9(9):Cd007491.
17. Facultad J, Lee GA. Patient satisfaction with a hospital-in-the-home service. Br J Community Nurs. 2019;24(4):179-85.
18. Levine DM, Pian J, Mahendrakumar K, Patel A, Saenz A, Schnipper JL. Hospital-Level Care at Home for Acutely Ill Adults: a Qualitative Evaluation of a Randomized Controlled Trial. J Gen Intern Med. 2021;36(7):1965-73.
19. Dismore LL, Echevarria C, van Wersch A, Gibson J, Bourke S. What are the positive drivers and potential barriers to implementation of hospital at home selected by low-risk DECAF score in the UK: a qualitative study embedded within a randomised controlled trial. BMJ Open. 2019;9(4):e026609.
20. Leff B, Burton L, Mader S, Naughton B, Burl J, Clark R, et al. Satisfaction with hospital at home care. J Am Geriatr Soc. 2006;54(9):1355-63.
21. Chua CMS, Ko SQ, Lai YF, Lim YW, Shorey S. Perceptions of Hospital-at-Home Among Stakeholders: a Meta-synthesis. J Gen Intern Med. 2022;37(3):637-50.
22. Chua CMS, Ko SQ, Lai YF, Lim YW, Shorey S. Perceptions of Stakeholders Toward "Hospital at Home" Program in Singapore: A Descriptive Qualitative Study. J Patient Saf. 2022;18(3):e606-e12.
23. Schulz R, Sherwood PR. Physical and mental health effects of family caregiving. Am J Nurs. 2008;108(9 Suppl):23-7; quiz 7.
24. Levine DM, Paz M, Burke K, Schnipper JL. Predictors and Reasons Why Patients Decline to Participate in Home Hospital: a Mixed Methods Analysis of a Randomized Controlled Trial. J Gen Intern Med. 2022;37(2):327-31.
25. Jeppesen E, Brurberg KG, Vist GE, Wedzicha JA, Wright JJ, Greenstone M, et al. Hospital at home for acute exacerbations of chronic obstructive pulmonary disease. Cochrane Database Syst Rev. 2012(5):Cd003573.

26. Shepperd S, Gonçalves-Bradley DC, Straus SE, Wee B. Hospital at home: home-based end-of-life care. Cochrane Database Syst Rev. 2021;3(3):Cd009231.
27. Gonçalves-Bradley DC, Iliffe S, Doll HA, Broad J, Gladman J, Langhorne P, et al. Early discharge hospital at home. Cochrane Database Syst Rev. 2017;6(6):Cd000356.
28. Edgar K, Iliffe S, Doll HA, Clarke MJ, Gonçalves-Bradley DC, Wong E, et al. Admission avoidance hospital at home. Cochrane Database Syst Rev. 2024;3(3):Cd007491.
29. Yu JJ, Sunderland Y. Outcomes of hospital in the home treatment of acute decompensated congestive cardiac failure compared to traditional in-hospital treatment in older patients. Australas J Ageing. 2020;39(1):e77-e85.
30. Cool L, Vandijck D, Debruyne P, Desmedt M, Lefebvre T, Lycke M, et al. Organization, quality and cost of oncological home-hospitalization: A systematic review. Crit Rev Oncol Hematol. 2018;126:145-53.
31. Shepperd S, Butler C, Cradduck-Bamford A, Ellis G, Gray A, Hemsley A, et al. Is Comprehensive Geriatric Assessment Admission Avoidance Hospital at Home an Alternative to Hospital Admission for Older Persons? : A Randomized Trial. Ann Intern Med. 2021;174(7):889-98.
32. Caplan GA. A meta-analysis of "hospital in the home". Med J Aust. 2013;198(4):195-6.
33. Wallis JA, Shepperd S, Makela P, Han JX, Tripp EM, Gearon E, et al. Factors influencing the implementation of early discharge hospital at home and admission avoidance hospital at home: a qualitative evidence synthesis. Cochrane Database Syst Rev. 2024;3(3):Cd014765.
34. Blom MC, Erwander K, Gustafsson L, Landin-Olsson M, Jonsson F, Ivarsson K. The probability of readmission within 30 days of hospital discharge is positively associated with inpatient bed occupancy at discharge--a retrospective cohort study. BMC Emerg Med. 2015;15:37.
35. Madsen F, Ladelund S, Linneberg A. High levels of bed occupancy associated with increased inpatient and thirty-day hospital mortality in Denmark. Health Aff (Millwood). 2014;33(7):1236-44.
36. JBI Manual for Evidence Synthesis: JBI; 2024 [Available from: https://synthesismanual.jbi.global/.
37. Peters MDJ, Marnie C, Tricco AC, Pollock D, Munn Z, Alexander L, et al. Updated methodological guidance for the conduct of scoping reviews. JBI Evid Synth. 2020;18(10):2119-26.
38. Tricco AC, Lillie E, Zarin W, O'Brien KK, Colquhoun H, Levac D, et al. PRISMA Extension for Scoping Reviews (PRISMA-ScR): Checklist and Explanation. Ann Intern Med. 2018;169(7):467-73.
39. Peters MDJ, Godfrey C, McInerney P, Khalil H, Larsen P, Marnie C, et al. Best practice guidance and reporting items for the development of scoping review protocols. JBI Evid Synth. 2022;20(4):953-68.
40. Cochrane Handbook for Systematic Reviews of Interventions 6.5 ed. Higgins JPT, Thomas J, Chandler J, Cumpston M, Li T, Page MJ, et al., editors: Cochrane; 2024 August 2024.
41. Covidence systematic review software Melbourne, Australia: Veritas Health Innovation; [Available from: www.covidence.org.
42. Tricco AC, Lillie E, Zarin W, O'Brien KK, Colquhoun H, Levac D, et al. PRISMA Extension for Scoping Reviews (PRISMA-ScR): Checklist and Explanation. Ann Intern Med. 2018;169(7):467-73.
43. Popay J, Roberts H, Sowden A, Petticrew M, Arai L, Rodgers M, et al. Guidance on the Conduct of Narrative Synthesis in Systematic Reviews - A Product From the ESRC Methods Programme 2006 [1:[Available from: https://citeseerx.ist.psu.edu/document?repid=rep1&type=pdf&doi=ed8b23836338f6fdea0cc55e161b0fc5805f9e27.
44. World Medical Association Declaration of Helsinki: ethical principles for medical research involving human subjects. JAMA. 2013;310(20):2191-4.
45. Sygeplejerskers samarbejde i N. Ethical guidelines for nursing research in the Nordic countries. Revidert opptrykk ed: Typotequet; 1995.
46. Caplan GA, Sulaiman NS, Mangin DA, Aimonino Ricauda N, Wilson AD, Barclay L. A meta-analysis of "hospital in the home". Med J Aust. 2012;197(9):512-9.
47. Huntley AL, Chalder M, Shaw ARG, Hollingworth W, Metcalfe C, Benger JR, et al. A systematic review to identify and assess the effectiveness of alternatives for people over the age of 65 who are at risk of potentially avoidable hospital admission. BMJ Open. 2017;7(7):e016236.
48. Brody AA, Arbaje AI, DeCherrie LV, Federman AD, Leff B, Siu AL. Starting Up a Hospital at Home Program: Facilitators and Barriers to Implementation. J Am Geriatr Soc. 2019;67(3):588-95.

Supporting information

S1 PRISMA-P 2015 Checklist

Click here to access/download
Supporting Information
S1 PRISMA-P-2015 checklist protocol.docx

Mapping the organisational and interventional framework for patients admitted to Hospital-at-Home for acute illness – a scoping review protocol

Authors

Kristina Kock Hansen[1,2*], Maria Klitgaard Christensen[1,3], Christian Backer Mogensen[4,5] Peter Biesenbach[6], Jette Holt[7], Pia Lysdal Veje[8], Mette Elkjær[1,5], Caroline Moos[1,9]

Affiliations

[1]Research Center for Integrated Healthcare Region of Southern Denmark, Aabenraa, Denmark
[2]Department of Medicine, University Hospital of Southern Denmark, Lillebaelt Hospital, Vejle, Denmark

[3]Department of Clinical Research, Faculty of Health Sciences, University of Southern Denmark, Odense, Denmark

[4]Department of Emergency Medicine, University Hospital of Southern Denmark, Aabenraa, Denmark

[5]Department of Regional Health Research, University of Southern Denmark, Odense, Denmark.

[6]Department of Emergency Medicine, University Hospital of Southern Denmark, Esbjerg, Denmark

[7]Infectious Disease Epidemiology & Prevention, The National Center for Infection Control (CEI), Copenhagen S, Denmark.

[8]University College South Denmark, Department for Applied Health Science, Denmark

[9]Department of Clinical Research, University Hospital of Southern Denmark, Aabenraa, Denmark

***Corresponding author**

E-mail: kristina.kock.hansen2@rsyd.dk (KKH)

Abstract

Objective

This proposed scoping review aims to map the components of Hospital-at-Home (HaH) organisational structures and interventions for the acute care of adults.

Introduction

Healthcare systems face increased pressure due to ageing populations having multiple morbidities, leading to strains on hospitals. Hospitalization is associated with higher risks of adverse events, particularly among older individuals. The surge in hospital admissions for older adults necessitates prioritising resources and exploring alternative solutions. HaH has emerged as a promising intervention, showing favourable outcomes in patient satisfaction, stress reduction and quality of life. However, the success of HaH depends on various factors, including organisational collaboration, intervention complexity and patient demographics.

Inclusion criteria

Studies based on adults with an acute illness requiring emergency care and initial contact with an emergency department (<24 hours), out of hours doctor or general practitioner, under the medical responsibility of the hospital, will be included.

Methods

We expect that this proposed scoping review will identify Scandinavian HaH solutions that have been part of a research process. Peer-reviewed studies from the databases Medline, Embase and CENTRAL will be included. Studies focusing on acute HaH solutions delivered in patients' homes will be considered. Data extraction will include information on study and patient characteristics, organisational structures and intervention details as well as effect outcomes. This protocol has been registered with Open Science Framework (DOI: 10.17605/OSF.IO/WKE7X).

Introduction

The Scandinavian healthcare systems are under pressure with the unprecedented numbers of people reaching retirement age and living longer with multiple chronic illnesses. These demographic changes are putting pressure on hospitals [1, 2]. Older people experience healthcare systems as fragmented with gaps in coordination. Additionally, challenges in retaining and recruiting healthcare professionals worsens the situation. Furthermore, there is a political agenda to reduce healthcare costs by reducing number of hospital beds [3] or length of stay [4]. Hospitalization is associated with a higher risk of nosocomial infections, inactivity, stress and delirium, particularly in older people [5-7].

Hospital admissions for older people over 75 years have surged by 65% over the past decade [8, 9], and recent trends across Europe show a year-on-year increase in the number of patients with acute medical illnesses presenting to hospitals [3]. Studies have shown that periods of emergency department (ED) crowding are associated with increased inpatient mortality, length of stay and costs for admitted patients, with a higher probability of patients leaving the ED against medical advice or without being seen [8, 9]. For these reasons, there is a strong need to prioritise hospital resources and find alternative and innovative solutions.

One promising intervention is Hospital-at-Home (HaH) [10, 11]. HaH has been shown to reduce adverse events and distress associated with hospitalization of adults and ease the burden on hospitals [12]. The literature contains numerous definitions of HaH, leading to diverse descriptions of interventions tailored to a broad range of patient populations requiring treatment for various conditions. The composition of healthcare professionals delivering HaH care differs considerably [10, 13]. HaH programs encompass various services, including end-of-life care, early discharge or admission avoidance [3, 10]. HaH, as described in the literature, is characterised by services that differ in patient selection, clinical procedures

and operational structure [3, 10]. Evidence on HaH programs within the Scandinavian context is limited, as Scandinavia has a less developed tradition of implementing HaH services. Additionally, Scandinavian healthcare system is characterised by free, publicly funded hospitals available to all citizens through taxation, making it challenging to draw direct comparisons with HaH solutions in countries like USA or the UK.

Our study's research question was driven by Danish healthcare administrators request for evidence on HaH solutions that ensure safety and effectiveness for patients and healthcare professionals. While Scandinavia has recently implemented some initiatives focusing on home-based telehealth and HaH services, there is limited published research on these efforts. Moreover, the literature appears to lack a comprehensive overview of various HaH solutions, including key aspects such as organisational structures, patient demographics and designated responsibility of care [3].

Typically, patients (18+ years) receiving HaH care report high satisfaction levels [14-17]. They value the comfort of their home setting, a heightened sense of independence and control and improvements in physical activity, mood, sleep quality and social interactions [14, 18-21]. Both adult patients and caregivers report that HaH is beneficial as it provides dignified care while preserving the quality of life [22]. Additionally, it enables family members to participate in patient care, providing more robust emotional support during the recovery process [22], but it can be physically and mentally demanding to be a participating caregiver [23]

However, the success of HaH is challenged by the collaboration between different health organisations and sectors, the complexity of interventions and uncertainty about which patients can be offered care and who is responsible for it. A review from 2020 highlighted that there is a lack of prioritisation of patient-centred care, including improvement of multidisciplinary continuity of care in the HaH services [13]. Furthermore,

adults ≥21 years old have expressed concerns about a potential decline in the quality of care and access to help if their condition worsens [22]. This was reflected in a randomized controlled trial by Levine et al., where 157 (63%) adults declined participation in a HaH intervention [24]. Chua et al. also reported that many relatives were anxious about the extra responsibility of caring for their loved ones in the context of HaH [22].

Four Cochrane reviews have focused on the effects of HaH with varying interventions and patient populations [25-28]. Admission avoidance appears to be an effective alternative to hospitalisation for adults with chronic obstructive pulmonary disease (COPD) exacerbations [25] or palliative care requirements [26]. Research has suggested that avoiding admission by providing acute care in the home is equally efficient to hospital care for older patients with acute cardiac failure [29] or oncological populations [30]. Generally, studies that aim to avoid hospital admittance reduce patient stress, shorten the length of hospital stay and lower the cost of care [12]. Studies vary in reporting how HaH affects mortality [11, 25, 31, 32]. A newly published Cochrane review focusing on factors influencing the implementation of HaH emphasized that clear guidelines to assess whom HaH is suitable for, provision of a trained workforce and consistent communication between staff, patients and caregivers were important when developing a HaH program [33].

Many HaH reviews focus on specific illnesses among patients or alternatives to admission with broader interventions and often do not elaborate on the intricacies of the organisations, interventions or patient characteristics [2, 34, 35]. The extensive array of HaH descriptions complicates identifying specific components that influence the planning of a potential HaH solution. We lack a concise overview of the organisational structure of acute HaH programs, encompassing the roles and responsibilities of various healthcare sector personnel. Implementation innovators need additional insights into the patient criteria suitable for HaH care and the specific interventions feasible within home settings to design effective, patient and work safe acute HaH programs.

Alternative solutions are imperative in anticipation of the future strain on the healthcare system. This proposed scoping review aims to map Scandinavian organisational structures, interventions and describe population characteristics as well as effect outcomes of adult patients receiving HaH care for an acute medical illness that would otherwise require acute hospital inpatient care for a limited period.

Review question

How is acute HaH care organised, which acute interventions are provided to what type of patients and what are the measured effects?

Materials and methods

The JBI Manual will guide the proposed scoping review for evidence synthesis, including developing this protocol to reduce the risk of impromptu decision-making [36, 37]. The review will be reported according to the Preferred Reporting Items for Systematic Reviews and Meta-Analysis (PRISMA-ScR) [38]. This protocol has been uploaded to Open Science Framework (DOI: 10.17605/OSF.IO/WKE7X). The search strategy will use the PCC (Population, Concept and Context) typology [39].

Search strategy

Searches will be conducted using the databases Medline (Ovid), Embase (Ovid) and CENTRAL (Cochrane). The preliminary search strategy for Embase is illustrated in Table 1. The search strategy will be peer-reviewed by a university librarian specialist specialising in medicine and health.

Table 1. Preliminary search strategy for Embase

#	Query
1	exp Home Care Services/
2	Exp Hospitals/
3	Exp Hospitalization/
4	2 or 3

5	exp Hospital to Home Transition/
6	1 and 4
7	5 or 6
8	(hospital* adj3 home).ti,ab,kf.
9	(home care and (medical services or health services)).ti,ab,kf.
10	(home based adj (medical care or medical services or health services or health care or healthcare or care services or care program*)).ti,ab,kf.
11	home ward.ti,ab,kf.
12	home based program*.ti,ab,kf.
13	admission avoidance.ti,ab,kf.
14	avoid* admission*.ti,ab,kf.
15	(hospital* adj2 avoid*).ti,ab,kf.
16	(hospital* or unit? or ward? or institution*).ti,ab,kf.
17	1 and 16
18	(Home-based adj2 hospital-based).ti,ab,kf.
19	Home hospitali?ation.ti,ab,kf.
20	Virtual ward.ti,ab,kf.
21	Home treatment.ti,ab,kf.
22	Hospital-based home*.ti,ab,kw.
23	Domiciliary hospitali?ation.ti,ab,kf.
24	Advanced care at home.ti,ab,kf.
25	(Substitute adj3 inpatient).ti,ab,kf.
26	"hospital at home".ti,ab,kf.
27	outpatient care.ti,ab,kf.
28	outpatient treatment*.ti,ab,kf.
29	7 or 8 or 9 or 10 or 11 or 12 or 13 or 14 or 15 or 17 or 18 or 19 or 20 or 21 or 22 or 23 or 24 or 25 or 26 or 27 or 28
30	exp Emergency Medical Services/ or exp Emergency Service, Hospital/
31	exp Emergency Treatment/
32	emergency department.ti,ab,kf.
33	Emergency care.ti,ab,kf.
34	rapid response.ti,ab,kf.
35	same day emergency care.ti,ab,kf.
36	emergency health service*.ti,ab,kf.
37	emergency treatment.ti,ab,kf.
38	emergency service*.ti,ab,kf.
39	emergency patient*.ti,ab,kf.
40	exacerbat*.ti,ab,kf.
41	(acute* adj2 illness).ti,ab,kf.
42	(acute* adj2 assessment).ti,ab,kf.
43	(acute* adj2 service*).ti,ab,kf.
44	(acute* adj2 decompensat*).ti,ab,kf.
45	acute medical.ti,ab,kf.
46	Acute disease.ti,ab,kf.
47	30 or 31 or 32 or 33 or 34 or 35 or 36 or 37 or 38 or 39 or 40 or 41 or 42 or 43 or 44 or 45 or 46
48	29 and 46
49	Exp Scandinavia/
50	(Denmark or Faroe Islands or Finland or Greenland or Iceland or Norway or Sweden or Scandinavia or Nordic countries).ti,ab,kf.
51	49 or 50
52	48 and 51

Searching for studies from start of database to search date

The search will be developed with three facets – acute care, Hospital-at-Home and Scandinavia. Each facet will use standardised keywords from the database's controlled vocabulary thesaurus and free text words with the Boolean operator "OR". The three facets will be combined using the Boolean operator "AND". The Hospital-at-Home facet was inspired by Cochrane reviews that developed search strings for this concept, albeit in two different reviews [16, 27]. No restrictions will be applied to the search date to ensure the identification of all relevant literature [40]. Publication type will be restricted to peer-reviewed studies to ensure research of reasonable quality but is not restricted to studies based on design. We will consider experimental, quasi-experimental, observational and descriptive studies for inclusion, as well as qualitative studies that address our review questions. Forward citation searches of included studies will be performed using the Web of Science database to identify any additional literature. Grey literature searches will consist of searching in *clinicaltrial.gov* and searches of the official Scandinavian health department websites.

Inclusion and exclusion criteria

Definitions and inclusion/exclusion criteria of primary studies for this scoping review have been summarised in Table 2.

Table 2 Definitions, inclusion and exclusion criteria based on the PCC typology

	Definitions	Inclusion	Exclusion
Population	Emergency department patients<br>Definition: Patients with the onset of an acute medical illness requiring emergency care [3]	• Adults 18+ years<br>• Initial medical contact with an emergency department (<24 hours), out of hours doctor or a general practitioner	• Adults requiring care described as post-surgery, long-term or end-of-life
Concept	HaH<br>Definition: Treatment at home under the medical responsibility of a hospital with a service that manages acute illness at a level of severity or complexity that would require hospital admission if the intervention was unavailable [3].	• Interventions describing admission avoidance to hospital	• Care not provided at the patient´s residence<br>• Interventions described as post-discharge, early-discharge or self-care by the patient<br>• Individuals with only a single contact with healthcare providers, for example, mobile acute care affiliated with an ED, who do not require admission.

Context	Scandinavia	• Studies that describe HaH in Denmark, the Faroe Islands, Finland, Greenland, Iceland, Norway, Sweden, Scandinavia, Nordic countries	• Studies from countries outside Scandinavia

Study selection

After searching, all identified citations will be imported into Covidence [41]. Covidence is a web-based collaboration software platform that streamlines the production of systematic and other literature reviews. In Covidence, duplicates will be eliminated, and results will be screened independently for eligibility by two researchers in a two-step process – title/abstract screening and full-text screening. Studies not qualifying for inclusion at full-text screening will be excluded with justification. A third reviewer will be used to adjudicate any disagreements. The search findings and the study inclusion process will be illustrated in a flow diagram following the PRISMA extension for Scoping Review guidelines [42].

Data extraction

The extracted data will include the following information: author, year of publication, country, the aim of the study, research design, participant characteristics (sex, age, comorbidities), type of acute conditions, patients´ underlying illness, availability of social support, intervention description, the organisation responsible for delivering the service, the person responsible for coordinating the services, telehealth tools such as remote monitoring and the designated healthcare provider with patient responsibility. Although the focus of the review is organisational structures and interventions, study outcomes such as mortality, readmission, length of treatment, depression, anxiety, functional status, quality of life, patient and caregiver satisfaction, caregiver stress, cognitive status, morbidity or economic costs will also be extracted and presented in the results section.

Proposed data synthesis

Data will be presented in tabular form (Table 3) and a narrative synthesis [43] will be conducted, including: 1) systematic categorization of patient characteristics, organisational structures and interventions, 2) mapping of the HaH organisations, including key stakeholders and healthcare professionals with the treatment responsibility and 3) exploring similarities and differences between the HaH organisation models, to ensure replicability and clarity.

Table 3 Characteristics of included studies for HaH in Scandinavia

Source	Design and method	Reason for HaH admission	HaH patient characteristics *	Patient comorbiditi es (if any)	What is the intervention/ treatment/ HaH service in the home?	Who is involved in the inter-vention (the organisational structure)?	Who has the medical treatment responsibilit y?	Outcomes measured and results

*number of patients, mean age, % sex

Ethical consideration

Ethical approval was not required because the study does not include participants. The study is in accordance with the Declaration of Helsinki [44] and Ethical Guidelines for Nursing Research in the Nordic Countries [45].

Discussion

Healthcare systems face additional strain from an aging population living longer with many chronic conditions, exacerbating pressures on EDs, which are associated with adverse outcomes such as increased mortality and more extended hospital stays [1, 2, 8, 9]. Generally, there is a growing imperative to allocate resources efficiently while exploring innovative, sustainable and evidence-based solutions that ensure patient safety. HaH has demonstrated favourable outcomes regarding patient satisfaction, stress reduction, prevention of hospital-acquired infections and reduced healthcare costs [10, 11].

If HaH offers the same treatment as in-hospital care, it will require significant equipment, technical devices and systems to monitor readmissions and record adverse events. It is essential to consider whether there can be similar access to services and diagnostic tools to what patients receive in the hospital [46]. Some literature describes the organisational structures of HaH programs as collaborations between hospitals and municipalities or specialised acute care teams [10, 47]. Challenges arise when multiple sectors need to collaborate on patient care and share resources [48]. Additionally, patients and healthcare professionals need to feel safe and confident about the treatment received in the home [13].

By limiting the literature to Scandinavian studies of admission avoidance, there is a risk of excluding important research and experiences from other countries or interventions, which may contribute to different perspectives or solutions. However, these limits ensure that HaH solutions are more homogeneous. The narrow grey literature search may have excluded relevant internal reports, white papers, conference papers, local initiatives or innovative pilot projects. However, these limitations were included to ensure we assessed the degree of research and the evidence base for Scandinavian HaH interventions.

We expect to present an overview of the components of Scandinavian HaH solutions. This review will benefit other health planners and researchers considering developing HaH solutions by focusing on examples of organisational structures and components of interventions in acute HaH care.

Author contributions

Conceptualization: KKH, CM, ME

Data curation: CM, KKH

Methodology: CM, KKH

Project administration: KKH

Validation: CM, KKH, MKC

Writing – original draft: KKH, CM

Writing – review & editing: KKH, CM, MKC, ME, CBM, PLV, JH, PB

Guarantor of the review protocol: Kristina Kock Hansen

References

1. Cho SJ, Stout-Delgado HW. Aging and Lung Disease. Annu Rev Physiol. 2020;82:433-59.
2. World Health Organsiation. Ageing 2018 [Available from: https://www.who.int/news-room/facts-in-pictures/detail/ageing.
3. Knight T, Lasserson D. Hospital at home for acute medical illness: The 21st century acute medical unit for a changing population. J Intern Med. 2022;291(4):438-57.
4. Stevenson A, Deeny S, Friebel R, Gardner T, Thorlby R. emergency hospital admissions in England: which may be avoidable and how? . The Health Foundation. 2018.
5. Klevens RM, Edwards JR, Richards CL, Jr., Horan TC, Gaynes RP, Pollock DA, et al. Estimating health care-associated infections and deaths in U.S. hospitals, 2002. Public Health Rep. 2007;122(2):160-6.
6. Vasilevskis EE, Han JH, Hughes CG, Ely EW. Epidemiology and risk factors for delirium across hospital settings. Best Pract Res Clin Anaesthesiol. 2012;26(3):277-87.
7. Brown CJ, Redden DT, Flood KL, Allman RM. The underrecognized epidemic of low mobility during hospitalization of older adults. J Am Geriatr Soc. 2009;57(9):1660-5.
8. Bernstein SL, Aronsky D, Duseja R, Epstein S, Handel D, Hwang U, et al. The effect of emergency department crowding on clinically oriented outcomes. Acad Emerg Med. 2009;16(1):1-10.
9. Sun BC, Hsia RY, Weiss RE, Zingmond D, Liang LJ, Han W, et al. Effect of emergency department crowding on outcomes of admitted patients. Ann Emerg Med. 2013;61(6):605-11.e6.
10. Varney J, Weiland TJ, Jelinek G. Efficacy of hospital in the home services providing care for patients admitted from emergency departments: an integrative review. Int J Evid Based Healthc. 2014;12(2):128-41.
11. Arsenault-Lapierre G, Henein M, Gaid D, Le Berre M, Gore G, Vedel I. Hospital-at-Home Interventions vs In-Hospital Stay for Patients With Chronic Disease Who Present to the Emergency Department: A Systematic Review and Meta-analysis. JAMA Netw Open. 2021;4(6):e2111568.
12. Wong SY, Teo YN, Wo YJ, Lim CW, Lai YF. Distilling lessons from home hospital implementation: a narrative scoping review. Singapore Med J. 2024.
13. Wang X, Stewart C, Lee G. Patients' and caregivers' perceptions of the quality of hospital-at-home service: A scoping review. J Clin Nurs. 2024;33(3):817-38.
14. Levine DM, Ouchi K, Blanchfield B, Saenz A, Burke K, Paz M, et al. Hospital-Level Care at Home for Acutely Ill Adults: A Randomized Controlled Trial. Ann Intern Med. 2020;172(2):77-85.
15. Levine DM, Desai MP, Ross J, Como N, Anne Gill E. Rural Perceptions of Acute Care at Home: A Qualitative Analysis. J Rural Health. 2021;37(2):353-61.
16. Shepperd S, Iliffe S, Doll HA, Clarke MJ, Kalra L, Wilson AD, et al. Admission avoidance hospital at home. Cochrane Database Syst Rev. 2016;9(9):Cd007491.
17. Facultad J, Lee GA. Patient satisfaction with a hospital-in-the-home service. Br J Community Nurs. 2019;24(4):179-85.
18. Levine DM, Pian J, Mahendrakumar K, Patel A, Saenz A, Schnipper JL. Hospital-Level Care at Home for Acutely Ill Adults: a Qualitative Evaluation of a Randomized Controlled Trial. J Gen Intern Med. 2021;36(7):1965-73.
19. Dismore LL, Echevarria C, van Wersch A, Gibson J, Bourke S. What are the positive drivers and potential barriers to implementation of hospital at home selected by low-risk DECAF score in the UK: a qualitative study embedded within a randomised controlled trial. BMJ Open. 2019;9(4):e026609.
20. Leff B, Burton L, Mader S, Naughton B, Burl J, Clark R, et al. Satisfaction with hospital at home care. J Am Geriatr Soc. 2006;54(9):1355-63.
21. Chua CMS, Ko SQ, Lai YF, Lim YW, Shorey S. Perceptions of Hospital-at-Home Among Stakeholders: a Meta-synthesis. J Gen Intern Med. 2022;37(3):637-50.
22. Chua CMS, Ko SQ, Lai YF, Lim YW, Shorey S. Perceptions of Stakeholders Toward "Hospital at Home" Program in Singapore: A Descriptive Qualitative Study. J Patient Saf. 2022;18(3):e606-e12.
23. Schulz R, Sherwood PR. Physical and mental health effects of family caregiving. Am J Nurs. 2008;108(9 Suppl):23-7; quiz 7.
24. Levine DM, Paz M, Burke K, Schnipper JL. Predictors and Reasons Why Patients Decline to Participate in Home Hospital: a Mixed Methods Analysis of a Randomized Controlled Trial. J Gen Intern Med. 2022;37(2):327-31.
25. Jeppesen E, Brurberg KG, Vist GE, Wedzicha JA, Wright JJ, Greenstone M, et al. Hospital at home for acute exacerbations of chronic obstructive pulmonary disease. Cochrane Database Syst Rev. 2012(5):Cd003573.

26. Shepperd S, Gonçalves-Bradley DC, Straus SE, Wee B. Hospital at home: home-based end-of-life care. Cochrane Database Syst Rev. 2021;3(3):Cd009231.
27. Gonçalves-Bradley DC, Iliffe S, Doll HA, Broad J, Gladman J, Langhorne P, et al. Early discharge hospital at home. Cochrane Database Syst Rev. 2017;6(6):Cd000356.
28. Edgar K, Iliffe S, Doll HA, Clarke MJ, Gonçalves-Bradley DC, Wong E, et al. Admission avoidance hospital at home. Cochrane Database Syst Rev. 2024;3(3):Cd007491.
29. Yu JJ, Sunderland Y. Outcomes of hospital in the home treatment of acute decompensated congestive cardiac failure compared to traditional in-hospital treatment in older patients. Australas J Ageing. 2020;39(1):e77-e85.
30. Cool L, Vandijck D, Debruyne P, Desmedt M, Lefebvre T, Lycke M, et al. Organization, quality and cost of oncological home-hospitalization: A systematic review. Crit Rev Oncol Hematol. 2018;126:145-53.
31. Shepperd S, Butler C, Cradduck-Bamford A, Ellis G, Gray A, Hemsley A, et al. Is Comprehensive Geriatric Assessment Admission Avoidance Hospital at Home an Alternative to Hospital Admission for Older Persons? : A Randomized Trial. Ann Intern Med. 2021;174(7):889-98.
32. Caplan GA. A meta-analysis of "hospital in the home". Med J Aust. 2013;198(4):195-6.
33. Wallis JA, Shepperd S, Makela P, Han JX, Tripp EM, Gearon E, et al. Factors influencing the implementation of early discharge hospital at home and admission avoidance hospital at home: a qualitative evidence synthesis. Cochrane Database Syst Rev. 2024;3(3):Cd014765.
34. Blom MC, Erwander K, Gustafsson L, Landin-Olsson M, Jonsson F, Ivarsson K. The probability of readmission within 30 days of hospital discharge is positively associated with inpatient bed occupancy at discharge--a retrospective cohort study. BMC Emerg Med. 2015;15:37.
35. Madsen F, Ladelund S, Linneberg A. High levels of bed occupancy associated with increased inpatient and thirty-day hospital mortality in Denmark. Health Aff (Millwood). 2014;33(7):1236-44.
36. JBI Manual for Evidence Synthesis: JBI; 2024 [Available from: https://synthesismanual.jbi.global/.
37. Peters MDJ, Marnie C, Tricco AC, Pollock D, Munn Z, Alexander L, et al. Updated methodological guidance for the conduct of scoping reviews. JBI Evid Synth. 2020;18(10):2119-26.
38. Tricco AC, Lillie E, Zarin W, O'Brien KK, Colquhoun H, Levac D, et al. PRISMA Extension for Scoping Reviews (PRISMA-ScR): Checklist and Explanation. Ann Intern Med. 2018;169(7):467-73.
39. Peters MDJ, Godfrey C, McInerney P, Khalil H, Larsen P, Marnie C, et al. Best practice guidance and reporting items for the development of scoping review protocols. JBI Evid Synth. 2022;20(4):953-68.
40. Cochrane Handbook for Systematic Reviews of Interventions 6.5 ed. Higgins JPT, Thomas J, Chandler J, Cumpston M, Li T, Page MJ, et al., editors: Cochrane; 2024 August 2024.
41. Covidence systematic review software Melbourne, Australia: Veritas Health Innovation; [Available from: www.covidence.org.
42. Tricco AC, Lillie E, Zarin W, O'Brien KK, Colquhoun H, Levac D, et al. PRISMA Extension for Scoping Reviews (PRISMA-ScR): Checklist and Explanation. Ann Intern Med. 2018;169(7):467-73.
43. Popay J, Roberts H, Sowden A, Petticrew M, Arai L, Rodgers M, et al. Guidance on the Conduct of Narrative Synthesis in Systematic Reviews - A Product From the ESRC Methods Programme 2006 [1:[Available from: https://citeseerx.ist.psu.edu/document?repid=rep1&type=pdf&doi=ed8b23836338f6fdea0cc55e161b0fc5805f9e27.
44. World Medical Association Declaration of Helsinki: ethical principles for medical research involving human subjects. JAMA. 2013;310(20):2191-4.
45. Sygeplejerskers samarbejde i N. Ethical guidelines for nursing research in the Nordic countries. Revidert opptrykk ed: Typotequet; 1995.
46. Caplan GA, Sulaiman NS, Mangin DA, Aimonino Ricauda N, Wilson AD, Barclay L. A meta-analysis of "hospital in the home". Med J Aust. 2012;197(9):512-9.
47. Huntley AL, Chalder M, Shaw ARG, Hollingworth W, Metcalfe C, Benger JR, et al. A systematic review to identify and assess the effectiveness of alternatives for people over the age of 65 who are at risk of potentially avoidable hospital admission. BMJ Open. 2017;7(7):e016236.
48. Brody AA, Arbaje AI, DeCherrie LV, Federman AD, Leff B, Siu AL. Starting Up a Hospital at Home Program: Facilitators and Barriers to Implementation. J Am Geriatr Soc. 2019;67(3):588-95.

Supporting information

S1 PRISMA-P 2015 Checklist

Response to Reviewers

We would be grateful if you could please revise the manuscript to respond to the comments raised by the Academic Editor and reviewers, and resubmit your manuscript files online once the necessary revisions are complete. Please accompany this resubmission by a detailed rebuttal letter that includes a point-by-point response to the concerns raised by the reviewers and Academic Editor and details on the revisions carried out on the manuscript since its original submission. Please also submit a "Track Changes" version of the MS file in the File Inventory. You may submit some of the files provided in your appeal request if appropriate.

We suggest that you take particular care to justify the changes to the focus of your review since your last submission. One of the goals of Study Protocols is to increase transparency. Therefore, we would recommend an explicit statement within the manuscript detailing the reasons for changes to the methods whilst this protocol underwent review, and justifications for doing so.

Response: Thank you for considering our appeal request and the opportunity for our protocol to be reconsidered. We appreciate the opportunity to provide clarity to the issues raised. We have revised the manuscript according to the comments raised by the Academic Editor and reviewers and included a point-by-point response to the concerns raised. As suggested, we have focused specifically on a transparent explanation of the change in search strategy that narrowed references to Scandinavia. The comments from reviewers have also resulted in us specifically addressing the chosen "analytical" approach narrative synthesis.

Reviewer #1:

Dear editor and authors,
Thank you for the opportunity to review the revised version of this scoping review protocol on Hospital-at-Home (HaH). The authors have clarified their manuscript in a thorough manner with well-founded revisions based on the comments from the reviewer. One aspect has, however, been added to the revised version without being specified in the initial version of the manuscript, neither asked for by the reviewers. That is the narrow focus on the Scandinavian perspective, absent in the initial version. I see how the introduction has been revised according to the Scandinavian focus, but I was surprised of this specification of inclusion. I also see how this narrow focus might conflict with the broad focus on the time perspective of the reported research. Would the authors share their rationale for the Scandinavian specific focus?

Response: Yes, the Scandinavian perspective was added to the revised version of the protocol. Our stakeholders were only interested in a review of HaH within the Scandinavian region. Our research team discussed that studies identified from other healthcare systems were perhaps not useful as they could not be easily implemented into a Danish context. Furthermore, as the protocol was not yet published, we were of the opinion that we had flexibility to make refinements to ensure the review remained as comprehensive and relevant as possible. Therefore, this adjustment aimed to ensure that our findings could directly inform the development and implementation of acute HaH programs in a context relevant to Denmark. Denmark´s healthcare is characterized by universal access, strong integration between primary and secondary care similar to other Scandinavian countries.

We have carefully documented and justified all modifications to maintain transparency and integrity. We hope this explanation clarifies our rationale and demonstrates our commitment to maintaining methodological rigor.

Reviewer 1: I acknowledge the risk of selection bias if adding a time limit; however, the context of specialized hospital-based services has changed greatly the last some-thirty years, in addition to the specific application of HaH in these settings – with a supplement of digital homebased services or communication. I cannot see the consistence in the authors argumentation between a broad time and a narrow context, and I worry the scoping will be in-consistent and result in a less stringent scoping overview. I would be interested in the reflections of the authors in this matter.

We acknowledge that hospital-based services and the application of HaH programs have evolved significantly over the past three decades. However, we are still interested in capturing all relevant developments within Scandinavia. HaH is a relatively new area of focus in Scandinavia and we were interested in all studies regardless of their age. Narrowing the timeframe could risk excluding research describing the initial implementation of HaH and the thoughts behind it. We hope this explanation clarifies our rationale and demonstrates our commitment to producing a focused, comprehensive, and impactful review.

Reviewer #2:

The authors have provided an insufficient revision that raises concerns. A Scandinavian focus was not clear from the initial version, raising questions of the study's rigour.

Response: See response above to reviewer 1

Reviewer 2: It is entirely inappropriate to not present an analytic plan in a protocol. A brief mention of a "narrative summary" is inappropriate. If the authors will not assess bias and/or quality, they cannot adequately present a synthesis of the literature.

Response:

Thank you for raising this important point and offering us the opportunity to explain in more depth. We will elaborate on our rationale for not including bias and quality assessments in this letter and will in our manuscript incorporate a more in-depth explanation of narrative synthesis.

As the primary aim of this scoping review is to map the breadth and characteristics of HaH solutions, including patient populations, interventions and organizational structures, it aligns with established guidance that scoping reviews are not intended to synthesize or critically appraise evidence as a systematic review would. Instead, they aim to provide a descriptive overview to identify knowledge gaps and understand the extent of the available literature (Arksey & O'Malley, 2005).

The inclusion of bias and quality assessments is not a standard requirement for scoping reviews, as noted in the JBI guidance on scoping reviews (Aromataris et al. 2024). Scoping reviews often focus on descriptive mapping rather than synthesizing results or evaluating the certainty of evidence. For example, it is appropriate to extract and descriptively map results without attempting to assess the certainty or quality of individual studies (Munn et al., 2018). This approach is particularly relevant for our review, which seeks to understand variations in HaH models and the contextual factors, rather than to draw definitive conclusions about intervention effectiveness.

For this review, we have opted for a narrative synthesis approach to systematically map the data while maintaining transparency and clarity in our methods. Please see line 181-185 in the manuscript. To support our methodological choice, we refer to Popay et al.'s (2006) guidance on narrative synthesis in systematic reviews, which highlights that narrative synthesis is a robust and widely accepted method, particularly when handling diverse and heterogeneous evidence bases. The approach allows for a structured and transparent

synthesis of findings without requiring quality assessments to be conducted as part of the process. Moreover, our narrative synthesis will be conducted systematically, emphasizing consistent reporting of patient characteristics, intervention types and organizational models.

We hope this response adequately addresses your concerns and provides clarity on why our approach, without bias or quality assessments, is methodologically sound and consistent with best practices for scoping reviews. Thank you again for your insightful comments, which have allowed us to strengthen our protocol.

References

Arksey, H., & O'Malley, L. (2005). Scoping studies: towards a methodological framework. International Journal of Social Research Methodology, 8(1), 19–32. https://doi.org/10.1080/1364557032000119616

Aromataris E, Lockwood C, Porritt K, Pilla B, Jordan Z, editors. JBI Manual for Evidence Synthesis. *JBI; 2024. Available from: https://synthesismanual.jbi.global. https://doi.org/10.46658/JBIMES-24-01*

Munn, Z., Peters, M.D.J., Stern, C. et al. Systematic review or scoping review? Guidance for authors when choosing between a systematic or scoping review approach. BMC Med Res Methodol 18, 143 (2018). https://doi.org/10.1186/s12874-018-0611-x

Popay, Jennie et al. "Guidance on the conduct of narrative synthesis in systematic Reviews. A Product from the ESRC Methods Programme. Version 1." (2006).
