# Supplementary material for: Mapping the organisational and interventional framework for patients admitted to Hospital-at-Home for acute illness in Scandinavia – a scoping review protocol
Source: PLoS One. 2025 Aug 4;20(8):e0328645. doi: 10.1371/journal.pone.0328645 (PMC12321056; doi:10.1371/journal.pone.0328645)
Supplement: Attachment — Submitted filename: Response to Reviewers.docx [file pone.0328645.s003.docx]

**Response to Reviewers**

**Reviewer #1:**

Thank you for the opportunity to review the submitted manuscript outlining a protocol for a scoping review on hospital at home, and relevant interventional and organizational factors to address. The manuscript is clear and well written; however, some revisions might enhance the clarity and readability.

*Response: Thank you for your time and effort to improve our manuscript. We have considered your suggested revisions and would like to make the following changes after your comments. Please be aware that the line numbers we refer to in current response letter are line numbers from the unmarked version of the manuscript.*

Abstract:

Perhaps adding a sentence on the expected relevance and findings, to add some arguments as to why this scoping is important to carry out? Or add a sentence after the objective on what you expect to identify?

*Response:*

*Line 33-34: “We expect that this proposed scoping review will identify all Scandinavian Hospital-at-Home interventions that have been part of a research process.”*

Introduction:

I suggest expanding the first argument of the introduction, adding some details on why older people are a strain to the health systems to better lay the ground for the reader.

*Response:*

*We have expanded the first argument of the introduction with the following text*

*Line 41-46:”The Scandinavian healthcare systems are under pressure with the unprecedented numbers of people reaching retirement age and living longer with multiple chronic illnesses. These demographic changes are putting pressure on hospitals [1, 2]. Older people experience healthcare systems as fragmented with gaps in coordination. Additionally, challenges in retaining and recruiting healthcare professionals worsens the situation. Furthermore, there is a political agenda to reduce healthcare costs by reducing number of hospital beds [3] or length of stay [4].”*

Some statements in the introduction is left without a reference, please add when appropriate, such as in line 79-80, line 86-87. Please check through the manuscript for other claims left without reference and add accordingly. Further, it seems unclear whether the mentioned literature is regarding all patients, adults, older adults, children, etc. Please add some details to clarify in which patients the claims are found.

*Response:*

*Thank you for pointing that out. We have added relevant references in the text, line 57-64.*

*Furthermore, we have added details about which age groups of patients we are referring to:*

*Line 77: “Typically, patients (18+ years) receiving HaH care report high satisfaction levels [14-17].”*

*Line 79-80: “Both adult patients and caregivers report that HaH is beneficial as it provides dignified care while preserving the quality of life [22].”*

*Line 88-90: “Furthermore, adults ≥21 years old have expressed concerns about a potential decline in the quality of care and access to help if their condition worsens [22].”*

*Line 90-91: “This was reflected in a randomized controlled trial by Levine et al., where 157 (63%) adults declined participation in a HaH intervention [24].”*

*Line 95-96:* “*Admission avoidance appears to be an effective alternative to hospitalisation for adults with chronic obstructive pulmonary disease (COPD) exacerbations [25] or palliative care requirements [26].”*

Both line 88 and line 90 starts with however, please revise.

*Response:*

*Thank you. This is now rectified.*

To clarify the role of this protocol, consider adding “proposed” in line 117 “this proposed scoping review.”

*Response:*

*Thank you for this suggestion. We have made these changes throughout the manuscript.*

In the aim and review question it remains unclear whether you are investigating HaH for adults, or all populations, please revise

*Response:*

*Thank you for this suggestion. In line 116, we have specified much more clearly that we are interested in adult populations only.*

Methods:

I suggest the design to be explicit in the opening paragraph of the methods, followed by a reference and some details on the JBI scoping in which you have chosen. This might enhance the remaining methods, making it clear to the reader what to expect.

*Response:*

*Thank you for these clear suggestions. We have added relevant references and changed the methods section now to include following text:*

*Line 124-128: “The JBI Manual will guide the proposed scoping review for evidence synthesis, including developing this protocol to reduce the risk of impromptu decision-making [36, 37]. The review will be reported according to the Preferred Reporting Items for Systematic Reviews and Meta-Analysis (PRISMA-ScR) [38]. This protocol has been uploaded to Open Science Framework (DOI: 10.17605/OSF.IO/WKE7X). The search strategy will use the PCC (Population, Concept and Context) typology [39].”*

Please consider adding some context as to how you identified the research question. As this is a protocol, you have the space, and it is valuable for the reader to get insight into how you identified the aim and RQ

*Response:*

*Thank you for pointing out this out. We have included the following text in the introduction:*

*Line 64-75: “Evidence on HaH programs within the Scandinavian context is limited, as Scandinavia has a less developed tradition of implementing HaH services. Additionally, Scandinavian healthcare system is characterised by free, publicly funded hospitals available to all citizens through taxation, making it challenging to draw direct comparisons with HaH solutions in countries like USA or the UK.*

*Our study's research question was driven by Danish healthcare administrators request for evidence on HaH solutions that ensure safety and effectiveness for patients and healthcare professionals. While Scandinavia has recently implemented some initiatives focusing on home-based telehealth and HaH services, there is limited published research on these efforts. Moreover, the literature appears to lack a comprehensive overview of various HaH solutions, including key aspects such as organisational structures, patient demographics and designated responsibility of care [3].”*

Inclusion – please rephrase as you are not including adults, rather reports investigating adults? Please, use a concise language.

*Response:*

*Thank you. We have removed the text and made a table instead with inclusion and exclusion criteria (also based on your next comment), page 8-9.*

The structure from inclusion and up until search strategy seems based on the PCC-tool for building search strategies, without you citing it. And, it seems the inclusion/exclusion only regards the patients, while the concept and context is clarify aspects of relevance to the inclusion, but it is not clear. I would suggest revising this part of the methods to explicitly state all inclusion and exclusion criteria, including those mentioned later in page 9/ line 165-169. Now, the eligibility criteria and search are described somewhat mixed.

*Response:*

*Thank you for your input. We decided that it would be clearer, if the inclusion and exclusion criteria for each block were presented in table format. Please see Table 2, page 9. We have also included the relevant reference for the PCC tool (line 128). We hope the descriptions of the search and subsequent eligibility criteria are now clearer.*

Is the search strategy from Embase final? If not, I would add “preliminary” or something to suggest it might change.

*Response:*

*Thank you for this suggestion. Yes, it was preliminary and is added in line 132 and in the heading of Table 1. We have decided to add an extra block to ensure that we only include Scandinavian research. This search strategy is therefore being amended (see Table 1, page 6-7).*

Are you going to search in all databases without a time limit? Why/why not?

*Response:*

*We have decided not to apply a time limit to the database search. Therefore, all databases will be search without a time limit. The reason behind this is to ensure that we do not increase the likelihood of bias, by excluding relevant evidence. (reference: Higgins JPT, Thomas J, Chandler J, Cumpston M, Li T, Page MJ, Welch VA (editors). Cochrane Handbook for Systematic Reviews of Interventions version 6.5 (updated August 2024). Cochrane, 2024.)*

*We have included the following information:*

*Line 140-141: “No restrictions will be applied to the search date to ensure the identification of all relevant literature [40].”*

Please, add a reference to Covidence, or some more data on the tool

*Response:*

*Thank you for identifying this. We have added a reference (line 155).*

Line 177-178 on peer review of the search, please move to the section on the search and not on the eligibility screening

*Response:*

*Thank you for identifying this. We have now moved the sentence to the appropriate subheading (line 141).*

Please cite the PRISMA ScR

*Response:*

*Thank you. This reference is now added in line 126.*

Please add some justification as to why you are extracting and planning to present data on the effects of the HaH interventions, and if relevant, please add to the aim and RQ

*Response:*

*Thank you for pointing this out. We would like to get a picture of the research based behind Hospital-at-Home interventions in Scandinavia. It is useful when planning a similar intervention to look at the effect outcomes measured. Therefore, the following text has now been incorporated into the last sentence of the introduction*

*Line 114-117: “Alternative solutions are imperative in anticipation of the future strain on the healthcare system. This proposed scoping review aims to map Scandinavian organisational structures, interventions and describe population characteristics as well as effect outcomes of adult patients receiving HaH care for an acute medical illness that would otherwise require acute hospital inpatient care for a limited period.”*

Please add reference to Helsinki and the Ethical Guidelines for Nursing research in the Nordic countries.

*Response:*

*Thank you. These references are now added (line 183-184).*

I would suggest adding a section on the proposed data analysis/synthesis/presentation. How are you going to summarize the findings and how are these to be presented? Perhaps removing the section named results, as you do not have any, and the current text is better placed in the methods. Then, the following section can be “Discussion” outlining the anticipated findings and their expected relevance.

*Response:*

*Thank you for this. We have now removed the result section and have added a “Proposed data presentation” section with a table indicating how we want to present our results. See line 174-179, page 10.*

Discussion:

Please, increase the amount of citations to relevant work, or rephrase so the reader can understand what are your claims and what are research supported claims

*Response:*

*Thank you for the input. The discussion section has been updated with citations to support research-based claims. You can see these changes in the discussion section between line 187 and line 200.*

**Reviewer #2:**

There are some elements of a scoping review protocol that are missing (e.g., data analysis, appraisal, limitations). I have the following comments.

For clarity, I recommend formatting hospital at home as a term. Such as hospital-at-home or 'hospital at home'.

*Response:*

*Thank you. We have formatted again using Hospital-at-Home as the term.*

Materials and Methods: 'Extension for Scoping Reviews' is missing from the PRISMA-ScR title.

*Response:*

*Thank you. This has now been amended in line 125-126.*

Inclusion criteria: There may be some typos. Perhaps the first sentence is meant to be that "In the scoping review, we will include studies that report primary research with adults..."

*Response:*

*We have adjusted the inclusion and exclusion criteria after both reviewers´ comments and identified the typos and corrected them. We have had a native English speaker editing the protocol. See Table 2, page 8 and 9.*

Inclusion criteria: Since the Background made a compelling focus on older adults, why is this review for all adults?

*Response:*

*Very relevant question, thanks. We are interested in identifying any Scandinavian HaH and later assessing if they are relevant for older adults.*

Please add a section on how data will be analysed.

*Response:*

*Please see Line 178-179 (Table 3). We expect the data to be very heterogeneous so a narrative summary will be the most relevant.*

*(Reference: Popay, J., H. Roberts, A. Sowden, et al. 2006. “Guidance on the Conduct of Narrative Synthesis in Systematic Reviews.” A Product From the ESRC Methods Programme Version 1, no. 1: b92.https://citeseerx.ist.psu.edu/document?repid=rep1&type=pdf&doi=ed8b23836338f6fdea0cc55e161b0fc5805f9e27).*

Will bias and/or quality be appraised?

*Response:*

*As this is a scoping review, bias and quality will not be appraised, but may be relevant for any later systematic review.*

*(Reference: Munn Z et al*. *Systematic review or scoping review? Guidance for authors when choosing between a systematic or scoping review approach. BMC Med Res Methodol. 2018 Nov 19;18(1):143. DOI: 10.1186/s12874-018-0611-x).*

Please add a Limitations section.

*Response:*

*We agree that a limitation section would be relevant. We have added the following text to the discussion section:*

*Line 202-208: “By limiting the literature to Scandinavian studies of admission avoidance, there is a risk of excluding important research and experiences from other countries or interventions, which may contribute to different perspectives or solutions. However, these limits ensure that HaH solutions are more homogeneous. The narrow grey literature search may have excluded relevant internal reports, white papers, conference papers, local initiatives or innovative pilot projects. However, these limitations were included to ensure we assessed the degree of research and the evidence base for Scandinavian HaH interventions.”*
