# Supplementary material for: Mapping the organisational and interventional framework for patients admitted to Hospital-at-Home for acute illness in Scandinavia – a scoping review protocol
Source: PLoS One. 2025 Aug 4;20(8):e0328645. doi: 10.1371/journal.pone.0328645 (PMC12321056; doi:10.1371/journal.pone.0328645)
Supplement: Attachment — Submitted filename: Response to Reviewer.docx [file pone.0328645.s004.docx]

**Response to Reviewers**

**We would be grateful if you could please revise the manuscript to respond to the comments raised by the Academic Editor and reviewers, and resubmit your manuscript files online once the necessary revisions are complete. Please accompany this resubmission by a detailed rebuttal letter that includes a point-by-point response to the concerns raised by the reviewers and Academic Editor and details on the revisions carried out on the manuscript since its original submission. Please also submit a “Track Changes” version of the MS file in the File Inventory. You may submit some of the files provided in your appeal request if appropriate.**

**We suggest that you take particular care to justify the changes to the focus of your review since your last submission. One of the goals of Study Protocols is to increase transparency. Therefore, we would recommend an explicit statement within the manuscript detailing the reasons for changes to the methods whilst this protocol underwent review, and justifications for doing so.**

*Response: Thank you for considering our appeal request and the opportunity for our protocol to be reconsidered.* *We appreciate the opportunity to provide clarity to the issues raised. We have revised the manuscript according to the comments raised by the Academic Editor and reviewers and included a point-by-point response to the concerns raised. As suggested, we have focused specifically on a transparent explanation of the change in search strategy that narrowed references to Scandinavia. The comments from reviewers have also resulted in us specifically addressing the chosen “analytical” approach narrative synthesis.*

**Reviewer #1:**

**Dear editor and authors,
Thank you for the opportunity to review the revised version of this scoping review protocol on Hospital-at-Home (HaH). The authors have clarified their manuscript in a thorough manner with well-founded revisions based on the comments from the reviewer. One aspect has, however, been added to the revised version without being specified in the initial version of the manuscript, neither asked for by the reviewers. That is the narrow focus on the Scandinavian perspective, absent in the initial version. I see how the introduction has been revised according to the Scandinavian focus, but I was surprised of this specification of inclusion. I also see how this narrow focus might conflict with the broad focus on the time perspective of the reported research. Would the authors share their rationale for the Scandinavian specific focus?**

*Response: Yes, the Scandinavian perspective was added to the revised version of the protocol. Our stakeholders were only interested in a review of HaH within the Scandinavian region. Our research team discussed that studies identified from other healthcare systems were perhaps not useful as they could not be easily implemented into a Danish context. Furthermore, as the protocol was not yet published, we were of the opinion that we had flexibility to make refinements to ensure the review remained as comprehensive and relevant as possible. Therefore, this adjustment aimed to ensure that our findings could directly inform the development and implementation of acute HaH programs in a context relevant to Denmark. Denmark´s healthcare is characterized by universal access, strong integration between primary and secondary care similar to other Scandinavian countries.*

*We have carefully documented and justified all modifications to maintain transparency and integrity. We hope this explanation clarifies our rationale and demonstrates our commitment to maintaining methodological rigor.*

**Reviewer 1: I acknowledge the risk of selection bias if adding a time limit; however, the context of specialized hospital-based services has changed greatly the last some-thirty years, in addition to the specific application of HaH in these settings – with a supplement of digital homebased services or communication. I cannot see the consistence in the authors argumentation between a broad time and a narrow context, and I worry the scoping will be in-consistent and result in a less stringent scoping overview. I would be interested in the reflections of the authors in this matter.**

*We acknowledge that hospital-based services and the application of HaH programs have evolved significantly over the past three decades. However, we are still interested in capturing all relevant developments within Scandinavia. HaH is a relatively new area of focus in Scandinavia and we were interested in all studies regardless of their age. Narrowing the timeframe could risk excluding research describing the initial implementation of HaH and the thoughts behind it. We hope this explanation clarifies our rationale and demonstrates our commitment to producing a focused, comprehensive, and impactful review.*

**Reviewer #2:**

**The authors have provided an insufficient revision that raises concerns. A Scandinavian focus was not clear from the initial version, raising questions of the study’s rigour.**

*Response: See response above to reviewer 1*

**Reviewer 2: It is entirely inappropriate to not present an analytic plan in a protocol. A brief mention of a “narrative summary” is inappropriate. If the authors will not assess bias and/or quality, they cannot adequately present a synthesis of the literature.**

*Response:*

*Thank you for raising this important point and offering us the opportunity to explain in more depth. We will elaborate on our rationale for not including bias and quality assessments in this letter and will in our manuscript incorporate a more in-depth explanation of narrative synthesis.*

*As the primary aim of this scoping review is to map the breadth and characteristics of HaH solutions, including patient populations, interventions and organizational structures, it aligns with established guidance that scoping reviews are not intended to synthesize or critically appraise evidence as a systematic review would. Instead, they aim to provide a descriptive overview to identify knowledge gaps and understand the extent of the available literature (Arksey & O’Malley, 2005).*

*The inclusion of bias and quality assessments is not a standard requirement for scoping reviews, as noted in the JBI guidance on scoping reviews (Aromataris et al. 2024). Scoping reviews often focus on descriptive mapping rather than synthesizing results or evaluating the certainty of evidence. For example, it is appropriate to extract and descriptively map results without attempting to assess the certainty or quality of individual studies (Munn et al., 2018). This approach is particularly relevant for our review, which seeks to understand variations in HaH models and the contextual factors, rather than to draw definitive conclusions about intervention effectiveness.*

*For this review, we have opted for a narrative synthesis approach to systematically map the data while maintaining transparency and clarity in our methods. Please see line 181-185 in the manuscript. To support our methodological choice, we refer to Popay et al.'s (2006) guidance on narrative synthesis in systematic reviews, which highlights that narrative synthesis is a robust and widely accepted method, particularly when handling diverse and heterogeneous evidence bases. The approach allows for a structured and transparent synthesis of findings without requiring quality assessments to be conducted as part of the process. Moreover, our narrative synthesis will be conducted systematically, emphasizing consistent reporting of patient characteristics, intervention types and organizational models.*

*We hope this response adequately addresses your concerns and provides clarity on why our approach, without bias or quality assessments, is methodologically sound and consistent with best practices for scoping reviews. Thank you again for your insightful comments, which have allowed us to strengthen our protocol.*

***References***

*Arksey, H., & O’Malley, L. (2005). Scoping studies: towards a methodological framework. International Journal of Social Research Methodology, 8(1), 19–32.* [*https://doi.org/10.1080/1364557032000119616*](https://doi.org/10.1080/1364557032000119616)

*Aromataris E, Lockwood C, Porritt K, Pilla B, Jordan Z, editors.* JBI Manual for Evidence Synthesis*. JBI; 2024. Available from:* [*https://synthesismanual.jbi.global*](https://synthesismanual.jbi.global/)*.* [*https://doi.org/10.46658/JBIMES-24-01*](https://doi.org/10.46658/JBIMES-24-01)

*Munn, Z., Peters, M.D.J., Stern, C. et al. Systematic review or scoping review? Guidance for authors when choosing between a systematic or scoping review approach. BMC Med Res Methodol 18, 143 (2018). https://doi.org/10.1186/s12874-018-0611-x*

*Popay, Jennie et al. “Guidance on the conduct of narrative synthesis in systematic Reviews. A Product from the ESRC Methods Programme. Version 1.” (2006).*
