# Supplementary material for: Mapping the organisational and interventional framework for patients admitted to Hospital-at-Home for acute illness in Scandinavia – a scoping review protocol
Source: PLoS One. 2025 Aug 4;20(8):e0328645. doi: 10.1371/journal.pone.0328645 (PMC12321056; doi:10.1371/journal.pone.0328645)
Supplement: Attachment — Submitted filename: Response_to_Reviewers_auresp_3.docx [file pone.0328645.s006.docx]

**Response to reviewers**

Dear reviewers #2, #3, #4, #5,#6, #7, #8 and Editorial team,

Thank you for your valuable feedback on our scoping review protocol article. We truly appreciate the effort invested in providing these insights, which will strengthen the final review. In this response letter and revised manuscript, we have outlined how we have addressed your concerns and the points that needed extra clarification. While the revisions may seem complex (due in part to the involvement of multiple reviewers), we have tried to structure our responses to ensure each reviewer has their concerns explicitly addressed, with clear indications of the changes made to the manuscript. We have also highlighted criticisms that multiple reviewers brought to our attention. We have included the page and line numbers where the adjustments can be made but decided that this was most simple for the reviewers if it referred to the “clean” manuscript. We hope this simplifies the revision process.

2 Reviewer #2
  2.1 I **recommended rejection of a prior version of this manuscript because there was no analytic plan presented. Analytic plans are a requirement for scoping review protocols. There is still no analytic plan in the manuscript.**

Response: We appreciate your commitment to ensuring the methodological rigor of this scoping review protocol. We acknowledge your concern regarding the absence of an explicit analytic plan in our previous submission. Based on your feedback, we have included the following additions in the manuscript to outline more specifically our analytic approach (line 157-172, page 9). This comment is similar to Reviewer #6 (see 6.17)

*“We will systematically map and synthesize the data, categorize organizational descriptions, and iteratively develop organizational models based on key themes.* *All included studies and documents will be systematically reviewed to identify descriptions of organizational structures, interventions, and patient characteristics related to the research question. Data will be extracted and then presented in a table format to visualize the roles, responsibilities, and collaborative relationships of actors across different sectors, as well as relevant interventions and patient characteristics.*

*The mapping process will follow an iterative approach, where data are continuously assessed and revised as new insights emerge from the included studies. The mapped roles and relationships will be compared to identify commonalities and variations. Based on the mapping process, a synthesis of the identified organizational models will be developed. These models will be continuously refined through an iterative process informed by new findings and theoretical considerations. The models will be described narratively and illustrated visually.*

*The final models will be presented with a discussion of their strengths and weaknesses. Furthermore, they will be considered in relation to practical application and their relevance for the organizational frameworks of future HaH solutions.”*

3 Reviewer #3

**RE Manuscript Number PONE-D-24-27648R2: The authors have presented a protocol for a scoping review on Hospital-at-Home structures and interventions for adults. Please find my detailed comments below.**

3.1 **I was unable to review the protocol on OSF. The authors should note that the OSF registration needs to outline the key information of the protocol clearly and transparently. Simply registering the title may not provide enough context for other researchers. You may refer to this for further information:** [**https://osf.io/ym65x**](https://osf.io/ym65x)**.**

Response: Thankyou for bringing this to our attention. This is our first attempt with using the OSF platform and we were unaware that the upload had not worked as intended. We have now uploaded the 2 versions of the protocol as it has developed. The first version had a broad focus (did not include a Scandinavia block) and the second version that indicates our intention for a more narrow focus. Please see this link *DOI: 10.17605/OSF.IO/WKE7X)*for your information.

3.2 **The abstract's transition from a general objective to focus on Scandinavian Hospital-at-Home is abrupt. The authors need to clearly state their focus in their objective.**

Response: Thankyou for bringing the general, broad abstract to our attention. We agree the transition was very abrupt, too abrupt. We have adjusted the abstract, deleting and adding text and more clearly highlighting that the focus is within Scandinavia and our objectives for doing this. See highlighted text throughout the abstract (line 22-43, page 2). Reviewer 4 had comments of a similar nature (see 4.1)

*“Objective*

*This proposed scoping review aims to map the components of Hospital-at-Home (HaH) organisational structures and interventions for the acute care of adults in Scandinavia.*

*Introduction*

*Healthcare systems face increased pressure due to ageing populations having multiple morbidities, leading to strains on hospitals. Hospitalization is associated with higher risks of adverse events, particularly among older individuals. The surge in hospital admissions for older adults necessitates prioritising resources and exploring alternative solutions. HaH has emerged as a promising intervention, showing favourable outcomes in patient satisfaction, stress reduction and quality of life. Recent health reform in Denmark has clearly indicated a clear political interest in HaH solutions. This review wishes to map Scandinavian HaH organizational collaboration, intervention set-up and patient demographics to assist Scandinavian healthcare administrators in understanding the current HaH models.*

*Inclusion criteria*

*Scandinavian studies based on adults with an acute illness requiring emergency care and initial contact with an emergency department…..*

*Methods*

*Peer-reviewed studies from the databases Medline, Embase and CENTRAL will be included without time limitation to present day and grey literature identifying information about HaH from Ministry of Health websites and clinicaltrial.gov will also be considered. Data extraction will include information on study and patient characteristics, organisational structures and intervention details as well as the measured effect outcomes. This protocol has been registered with Open Science Framework (DOI: 10.17605/OSF.IO/WKE7X).”*

3.3 **The methods of the abstract need to outline the date range of the study. Furthermore, focusing only on peer-reviewed articles is not in line with the methodology of a scoping review.**

Response: Thank you for these reminders. As this is a protocol article, we are waiting for final feedback before setting the current date for the actual search. We have no plans to limit the search in relation to time. Databases will be searched from inception to the current date. We have adjusted this in the abstract as instructed. We have chosen to focus only on peer-reviewed articles from the health databases as we expect that it is improbable that other types of articles in these databases (eg.conference abstracts) include detailed organization descriptions. However, we agree that scoping reviews should examine grey literature, and we have chosen to look at health ministry websites in Scandinavian countries and the ClinicalTrials.gov database. We have included this information in the methods section in the abstract (line 40-41, page 2).

*“Peer-reviewed studies from the databases Medline, Embase and CENTRAL will be included without time limitations and grey literature will be searched from Ministry of Health websites and ClinicalTrials.gov.”*

3.4 **The consistency and cohesion between the topics presented in the introduction needs to be revised. For example, after the paragraph discussing the origin of the research question, readers might expect a more direct continuation of that theme. However, the subsequent paragraphs introduce a new concept, which could disrupt the flow of the introduction.**

Response: Thankyou for this constructive feedback. There have been a number of other reviewers that have commented on the flow of the introduction (See reviewer 6.4 and 8.1). We have made a number of changes to improve this focusing on the flow of topics. Please see line 45-79, page 3-4.

3.5 **The review question needs to implement the PCC framework. At the time of writing this peer review, the review question is rather broad and difficult to navigate and understand. The authors may consider reformulating the research question in three distinct segments.**

Response: We concur and using the PCC framework have reworded the research question. Please see line 81-83, page 4 and comments from reviewer #6.8

*“How are HaH interventions for patients requiring emergency care organized in Scandinavia? What are the characteristics of the patients offered HaH care?”*

3.6 **I concur with the reviewers and editor that the focus on Scandinavian region may be too narrow for the purpose of a scoping review. However, since the authors are interested only in the Scandinavian region, I would recommend that they target the grey literature that are more relevant to that specific region. This source may provide useful databases that the author may consider to search and state in their protocol:** [**https://www.hvl.no/en/library/find-databases-and-other-resources/**](https://www.hvl.no/en/library/find-databases-and-other-resources/)**.**

Response: Thankyou for this suggestion. We referred the question of grey literature to our medical information specialist and decided, collaboratively, that ClincalTrials.gov and websites from health authorities was adequate in relation to our time and resources. Our stakeholders are primarily interested in published research in the field but could see the value of a limited grey literature search.

3.7 **I would recommend the authors to summarize their data extraction in a table and present them to the readers. This will also save their time when extracting the results in the review process once you have preplanned data extraction table. The authors can then use the freed space in their protocol to articulate on how they will organize the data extraction. They can elaborate the process on data extraction, namely the minimum number of people involved, and how disagreements will be resolved.**

Response: We appreciate your suggestion to include a preplanned data extraction table in our manuscript to improve clarity and efficiency in the review process. Reviewer 4.1 and 5.4 had similar concerns. As suggested, we have altered the data charting paragraph with the inclusion of Table 3 and Table 4 (see line 153-156, page 8-9) and expanded on the extraction process. We have also altered the data extraction heading to data charting process according to reviewer #4’s request (see 4.2 for changes in text and line 142-145, page 8 in the manuscript:

*“The data charting process will be carried out using three researchers. One researcher (first author) will extract all data from all papers. This other two researchers will extract data blinded from the first author and any disagreements will be resolved by discussion.*

**3.8 Instead of the title 'Proposed data synthesis' the authors may consider 'Data analysis'.**

Response: We have made this change as requested (see line 157, page 9).

**3.9 I concur with the authors regarding risk of bias assessment being inappropriate in the context of a scoping review. Still, I would encourage the authors to include a statement in their protocol that they will not critically appraise the literature using any risk of bias assessment tools.**

Response: Thankyou for your support with our assessment of relevance. We have included the following sentence under data analysis in the methods section (see line 172-173, page 9):

*“We will not critically appraise the literature using risk of bias assessment tool, rather, all relevant studies will be included.”*

**3.10 Under the Discussion section, the authors have stated that by limiting the results to Scandinavian studies, the output will be homogenous. I would argue that some readers may misinterpret this from a statistical perspective, as in the homogeneity of data will allow the authors to conduct certain statistical analysis. I would recommend the authors using other terminologies to discuss this shortcoming and link this to what was expected from the Danish healthcare administrators.**

Response: Thankyou for this comment. We agree that the word “homogenous” is often used with quantitative data in a statistical analysis. We have changed the word to “comparable” (line 198, page 10).

**3.11 I would suggest the authors clearly state their dissemination plans once the review is over. This could include presentation of the findings in a peer-reviewed journal.**

Response: Thanks for your suggestion. We have added the following to the manuscript (line 211-214, page 11):

*“The review will be submitted for publication in a Scandinavian peer-reviewed journal and will be shared through institutional websites and LinkedIn. If possible, the preliminary results will be presented at an international conference”*

**3.12 PRISMA-P was not designed to report scoping review protocols. I would recommend the authors delete that in case of future submissions.**

Response: Thanks for drawing this to our attention. We have been a little in doubt what reporting checklist to follow as there is no PRISMA extension for scoping review protocols as you have pointed out and PRISMA-ScR is used for the scoping review itself not the protocol article. PLOS one has a journal requirement that the PRISMA-P checklist is used and included.

4 Reviewer #4

**Dear Respectable Authors
Thank you for considering a great area of research related to acute illness.**

**4.1 Your responses to previous reviewer is well but your manuscript needs some revisions as follows; - Abstract, methods section, please add some information regarding charting methods.**

Response: Thanks for pointing out that information regarding charting methods should be added to our manuscript. Many of these issues have been address with other reviewers (see 3.7 and 5.4). Please see response to 3.2 (Abstract) and 3.7 (Methods and Data charting). See line 142-156, page 8-9 in the manuscript.

**4.1 Based on the PRISMA-ScR, the eligibility criteria is the first step in reporting a scoping review. Please set this subheading as the first item in the methods section, after protocol and registration and then mention the search.**

Response: Thankyou for identifying this improvement. PLOS one’s journal requirement request PRISMA-P checklist. As both PRISMA-P and PRISMA –ScR have eligibility criteria as the first step in the methods we have moved the heading as suggested. See line 92-97, page 4-5.

**4.2 In scoping review we use the "data charting process" instead on "data extraction", then, it is better to use a separate subheading including "data charting process and data items" (Items 10 and 11 of PRISMA ScR).**

Response: Thank you for your feedback. We acknowledge the useful distinction between "data extraction" and "data charting process" in scoping reviews and agree that it can be used in the manuscript.

5 Reviewer #5

**This is a useful topic and appropriately justified. I understand the limit to the Scandinavian perspective if the research is being undertaken to satisfy particular stakeholders. I believe the methods that are selected are appropriate and have been adequately justified, particularly with regard to the time span and setting**

Reponse: Thankyou for this feedback. Our stakeholders were solely interested in Scandinavian literature and although we explored broadening the search to start with – this was not possible logistically and did not fit with the aims of the stakeholders. Therefore the third block was added. We had understood that as a protocol article changes would be acceptable up until publication but can see that this has added confusion, unfortunately. See comments also from reviewer #3 and #6.

**5.1 That said, more detail and transparency is needed for the literature search. It is not clear how exactly the peer review will be undertaken (PRESS peer review** [**https://pubmed.ncbi.nlm.nih.gov/27005575/**](https://pubmed.ncbi.nlm.nih.gov/27005575/) **is the most commonly used method) and how the authors commit to addressing any concerns.**

Response: Thankyou for the opportunity to address this issue. The search strategy for Embase was peer-reviewed by a information specialist specialising in medicine and health. This review involved the first author sending the “primary search strategy” from Embase to the the information specialist. The search strategy was fine-tuned using suggestions from the PRESS checklist over a couple of weeks with issues addressed in collaboration. We have added the following information to the manuscript (see line 102-105, page 5)

*“The first author sent the “preliminary search strategy” derived from Embase to the information specialist. The search strategy was fine-tuned using suggestions from the PRESS checklist over a couple of weeks with changes made collaboratively. There was specific feedback given to the use of Boolean and proximity operators as well as database specific subject headings.”*

**5.2 Some errors or limitations of the current strategy are noted - line 48 should combine lines 29 and 47, not 29 and 46. There are changes in language and truncation that would also enhance the strategy. For example, the geographic filter doesn’t incorporate regional areas, capital cities, citizens (e.g., Danish, Norwegian) and there are alternate spellings for some locations such as the Faeroe Islands. It would also be helpful to see the number of hits per line. There is a small error in reference to the grey literature source, which is ClinicalTrials.gov (i.e., plural). MEDLINE should be written in block caps.**

Response: Thankyou for noting these errors. We agree that line 48 contained a typing error and is now corrected (see line 51, page 7 in the search strategy for Embase). But we have included the suggestions you made in relation to truncations to include citizens and have include alterations to alternative spellings such as the Faeroe Islands. We have now included the adjusted search and the number of hits per line. The other small typographical errors you identified are also identified. We don’t believe that addition of regional areas and capital citites is possible systematically throughout all of Scandinavia. See line 107, page 6-7 for the adjusted search strategy.

**5.3 The authors state that deduping will be done within Covidence. If work is not yet complete, I would recommend using a reference manager software such as EndNote for initial deduping, with final/refined deduping done in Covidence. It is challenging to confirm duplicates within Covidence compared to the flexibility available in most reference manager software.**

Response: Thank you for the thoughts on deduplication. We would prefer to continue using Covidence but are aware that the flexibility is not as good. We base this decision on the reference below. Although this reference considers systematic reviews and this is a scoping review the findings are applicable and support our decision to deduplicate with Covidence.

McKeown, S., Mir, Z.M. Considerations for conducting systematic reviews: evaluating the performance of different methods for de-duplicating references. Syst Rev 10, 38 (2021). https://doi.org/10.1186/s13643-021-01583-y

**5.4 I am not clear who is doing the data extraction. This should be stated in the methods.**

Response: Thankyou for highlighting this error. Other reviewers (see #3.7 and 4.1) have also requested this information. Please see response to reviewer 3.7 and line 142-145, page 8 in the manuscript. This is now included in the methods.

**5.5 There is an inaccuracy in the example regarding HaH solutions, where the authors suggest that it is challenging to compare the Scandinavian system with that of the UK. I believe the UK healthcare system and its hospitals are publicly funded, so another example/country alongside the US should be highlighted.**

Response: Thank you for your feedback. We acknowledge the inaccuracy in our example regarding HaH solutions and the comparison between the Scandinavian and UK healthcare systems. You are correct that the UK healthcare system is publicly funded, making it more comparable to Scandinavian models. To improve clarity and accuracy, we will revise our example and have added Singapore, as they have different funding structures that may provide a more relevant contrast. We appreciate your input and have made following changes in the manuscript (line 70-72, page 4):

*“The Scandinavian healthcare system is characterised by free, publicly funded hospitals available to all citizens through taxation, making it challenging to draw direct comparisons with HaH solutions in countries like USA or Singapore.”*

**5.6 The manuscript flows and is easy to understand although some sentences are a bit stilted, no doubt because the authors are writing in their non-native tongue (for which they are to be congratulated). E.g., Line 51 – “political agency to reduce healthcare costs by reducing number of hospital beds…” requires “the” before number; Line 71 – “Additionally, Scandinavian healthcare system is characterised…” requires “the” before Scandinavian. Line 118 is also a bit unclear – “to design effective, patient and work safe acute HaH programs” – suggest instead wording as “to design effective HaH programs that are safe for both patients and providers (if that is the intended meaning). I would suggest some small copy edits in the manuscript (e.g., mathematical symbols should be written as text in the manuscript, so change “<” to less than, “18+” to 18 years of age and older (or something similar, e.g., patients over the age of 18). “Adults >= 21 years old” should be written as “Adults aged 21 or older”. In the abstract, “out of hours” should be hyphenated for “out-of-hours doctor or general practitioner”. There is a possessive missing at the end of administrators’ in line 76 “driven by Danish healthcare administrators request”. I would substitute “although” for “but” in the line “it can be physically and mentally demanding… Free text should be hyphenated in the line “free-text words with the Boolean operator…”.**

Response: Thank you for your thorough and constructive feedback. We have revised the manuscript to address the grammatical issues you pointed out. Additionally, mathematical symbols have been written in text form and we have incorporated your recommendations for phrasing in the manuscript.

6 Reviewer #6

**Dear Authors and Editor,**

**Thank you for considering me as a reviewer for this manuscript titled “Mapping the organisational and interventional framework for patients admitted to Hospital-at-Home for acute illness – a scoping review protocol.” Below are my observations:**

**General Comments**

**I understand that this is the third (?) round of comments on this manuscript, which is a scoping review protocol. I have read the previous reviewers’ comments as well as the authors’ responses. I understand that the initial version of the manuscript did not specify a contextual framework (Scandinavian), but the authors later modified this aspect in the subsequent versions. On this point, I agree with the authors regarding the need to reassess the protocol and adapt it to the research needs. However, it is important to understand the editorial process, as reviewers evaluate an initial version and provide comments based on that version. When modifications are made that were not requested, this can create confusion, as it complicates the understanding of what the authors actually intend to investigate. That said, regarding the restriction to the Scandinavian context, the authors should consider the following:**

Response: Thank you for your thoughtful feedback and for taking the time to review both our manuscript and the previous rounds of comments. This response is similar to reviewer #3.6 and #5. Please see our general comments to the reviewer. We understand your concerns regarding modifications made in response to reviewer feedback and the potential confusion it may have caused in the editorial process. Our intention in refining the protocol to focus on the Scandinavian context was to ensure a more precise and relevant scope for our research. We acknowledge that changes beyond direct reviewer requests should be clearly justified, and we appreciate the opportunity to clarify our approach. We are aware of your request to align the manuscript more closely with the JBI Evidence synthesis manual. We have tried to do this as requested in the responses to your suggestions.

**6.1. In the introduction, the authors mention that research on this topic in the Scandinavian context is limited. Would this not contradict the decision to limit the review exclusively to the Scandinavian context? Additionally,**

Response: Thank you for the question. The decision to focus exclusively on the Scandinavian context was driven by the scope and priorities set by our stakeholders. Our aim is to provide insights that are directly applicable to the healthcare systems in Scandinavia, where structural and organizational factors may differ significantly from other healthcare systems. While research on this topic is limited in Scandinavia, this gap in knowledge is precisely why our stakeholders emphasized the need for a targeted review. By focusing on this specific context, we aim to generate findings that are both relevant and actionable for policymakers and healthcare providers in the region.

**6.2. Why is the Danish healthcare system so different from other systems that it could not benefit from research produced in other contexts? If research in the Scandinavian context is limited, knowing what is happening beyond this context could provide the authors with a more comprehensive overview of the strategies being used elsewhere and how they could be implemented in their setting.**

Response: The decision to focus on Scandinavia specifically stems from a strong belief among our stakeholders that the Scandinavian healthcare system has distinctive characteristics that may not fully align with other international systems. These differences, including historical, cultural, and policy-driven factors, mean that lessons and strategies from other countries may not always translate effectively to the Danish context without adaptation. That said, we agree that understanding international research and strategies is valuable. Our study aims to build a solid foundation in the Scandinavian context. However, our primary goal is to ensure is to map the research that exists already from Scandinavia to see how directly applicable it is to Denmark.

**6.3. The authors could consider formulating a sub-question to address the Scandinavian context specifically, allowing the main research question to remain open and better aligned with the scoping review methodology.**

Response: Thank you for your suggestion regarding our research question. Our primary objective in this review is to synthesize a comprehensive description of the organizational framework of HaH models within a Scandinavian context. By focusing specifically on this, we aim to provide an overview of the varying models across Scandinavian countries, allowing for a deeper understanding of their similarities, differences and how they operate in these settings. While we recognize that a broader view on HaH solutions, e.g. from the UK, may provide useful insights, we believe that directly applying it to the Scandinavian context would be challenging due to the differences in healthcare systems, policies and organizational structures. As such, we aim to focus on what is specific to Scandinavia to offer relevant and actionable insights within this particular context. A search using the two blocks, HaH and emergency care, initially resulted in many relevant hits. However, it became clear that assessing the organizational frameworks of HaH interventions required full-text reading. Due to practical and organizational constraints, as well as reduced interest from our stakeholders in systems outside Scandinavia, the inclusion of a third block was deemed necessary.

**6.4 Introduction. The introduction is appropriate; however, there are pieces of information that appear disconnected from the text or are not discussed in context. For example: Lines 52-53: “Hospitalization is associated with a higher risk of nosocomial infections, inactivity, stress and delirium, particularly in older people [5-7].” Additionally, in the introduction, the authors frequently refer to the older adult population; however, the study will include individuals aged 18 and over. I suggest clarifying this point.**

Response: Thank you for your feedback. thanks for these comments. There are other reviewers that wanted us to reconsider the introduction. See reviewer 3.4 and 8.1. We have completely revised the introduction and included the suggestions you offer here.

**6.5 The authors mention (lines 51-52) that there are policies aimed at reducing the number of hospital beds and the length of stay. In this regard, the potential of HaH as an alternative is not adequately discussed, especially considering that a more complex logistical structure is required to provide hospital-level care at home, particularly in cases of acute illness (as proposed in this review).**

Response: Thank you for highlighting this important point. We appreciate your careful review of our manuscript. Regarding your comment we acknowledge that the potential of HaH as an alternative to traditional hospitalization could be further elaborated. Specifically, as you rightly point out, providing hospital-level care at home, particularly for acute illness, does require a more complex logistical structure. We agree that this aspect is crucial to understanding the feasibility and challenges of implementing HaH models. Therefore, we have added following text to the manuscript (line 55-56, page 3):

*“However, a more complex logistical structure is required to provide hospital-level care at home, particularly in cases of acute illness”.*

**6.6 Another point that caught my attention, which I suggest reviewing, is citation [29] referring to “older patients with acute cardiac failure.” While the information is accurate, upon reviewing the cited article, it appears to be a retrospective study. Given the methodological limitations of retrospective studies, it may not be sufficient to generalize the findings to the broader population. Care should be taken when using individual studies to make generalizations, especially when there are limitations in their design. For instance, if we consider an older adult with decompensated heart failure, we should question whether, in an acute situation, they would receive better care at home than in a hospital. This is particularly relevant in healthcare systems with limited financial and human resources. Therefore, while the information is not incorrect, it requires further discussion.**

Response: Thank you for your thoughtful feedback on the reference to citation [29] regarding "older patients with acute cardiac failure”. After careful consideration, we agree with your point that the generalization of findings from retrospective studies can be problematic, particularly in the context of acute conditions like decompensated heart failure. Given the complexities of providing hospital-level care at home for such patients, we recognize the need for more careful discussion of these factors. To address this concern, we have decided to remove the sentence and reference [29] from the manuscript. This will help avoid potential misinterpretation.

**6.7 Methods: The authors state that the protocol development adhered to the PRISMA-ScR guidelines (correct); however, the attached document refers to PRISMA-P, which is confusing. Is there an explanation for this? If not, PRISMA-ScR should replace PRISMA-P.**

Response: Thank you for allowing us to address this comment. We believe the confusion arises from the fact that there is no specific checklist for scoping review protocols—only for systematic review protocols (PRISMA-P). While there is a PRISMA extension for scoping reviews (PRISMA-ScR), PLOS One’s journal requirements request authors to use PRISMA-P despite it being a scoping review protocol. Hence the confusion. See also reviewer 3.12 and Reviewer 4.1

**6.8 The PCC acronym is central to scoping reviews; therefore, its structure should be consistently reflected throughout the methodology section. How is PCC integrated into the search strategy?**

Response: Thank you for the opportunity to clarify this question. Reviewer #3.5 had similar comments. Although, Table 1 explains this in detail it was unclear in the wording of the research question. So in accordance with comments from other reviewers we have reworded the research question to better reflect the PCC typology and search strategy. Population: patients requiring acute care; Concept: Hospital at home; Context: Scandinavia (see line 83-85, page 4).

**6.9 Additionally, why are only three databases included? While Cochrane recommends these three as essential, in scoping reviews, the goal is to map the phenomenon extensively. Therefore, it is advisable to include additional databases (e.g., Scopus, Web of Science, CINAHL). I suggest reconsidering this. Similarly, regarding grey literature—why is only one database (clinical trials) included?**

Response: Thank you drawing this to our attention. We included Web of Science in relation to citation searching and will look at department websites as part of the grey literature search. The limited number of databases reflects the limited time and resources given to this project from our stakeholders. There was also limited interest from our stakeholders in grey literature. They were interested in HaH solutions in Scandinavia that has published research results connected to it.

**6.10 Please add a mention of the controlled vocabularies that will be used.**

Response: This is mentioned in line 111-112, page 7:

*“Each facet will use standardised keywords from the database´s controlled vocabulary thesaurus…..”*

**6.11 For articles that are not fully accessible, what is the plan to obtain them?**

Response: Article accessibility is not considered problematic and those we cannot find can be ordered through the university*.*

**6.12 Considering the limited existing evidence, do the authors believe that this will be adequately reported in the studies? If not, how will they evaluate this aspect: "Initial medical contact with an emergency department (<24 hours), out-of-hours doctor, or a general practitioner" (line 158)?**

Response: We believe that HaH often has many definitions and it was important that we looked at studies with a similar definition of this concept. We were not interested in early discharge models of HaH, only admission avoidance (stakeholder decision). We believe that although there may be limited evidence it was important to have decided *a priori* on a definition of the concept. Therefore, this criteria was considered necessary and articles were only included if they met this specific definition of HaH.

**6.13 In the study selection process, I suggest adding information on the agreement between reviewers before starting the screening process. Please refer to the relevant section in the JBI Manual.**

Response: Thankyou for this comment. We have added the following to the manuscript (line 139-140, page 8):

*“All disagreements regardless of selection stage will be resolved by consensus in accordance with the JBI Manual for Evidence Synthesis [20].”*

**6.14 Lines 166-167: “The search findings and the study inclusion process will be illustrated in a flow diagram following the PRISMA extension for Scoping Review guidelines.” This is incorrect. There is no extension of the PRISMA flow diagram for scoping reviews. The flow diagram is the same for all types of reviews; what changes is the checklist. Please revise this.**

Response: Thank you for identifying this error. We have removed the words “extension” and the referral specifically to scoping reviews (line 140-141, page 8):

*“The search findings and the study inclusion process will be illustrated in a flow diagram following the PRISMA guidelines [21].”*

**6.15 How will the grey literature be reviewed?**

Response: Thankyou for the opportunity to expand on this process. We have included the following to the manuscript (line 121-124, page 7):

*“Grey literature from ClinicalTrials.gov will be imported into Covidence and screened with double-blinding. One author will screen any relevant website information and if considered relevant the information will be screened by a second author.”*

**6.16 How are the extraction variables related to the review topic? How were they chosen? Did the authors adapt the extraction form from other studies, or was it created specifically for this review? Please consult the JBI Manual.**

Response: Thank you for allowing us to elaborate on this point. The data charting form was developed specifically for this review by the first author. The data charting forms can be seen in Table 3 and 4 and we expand on this point in our response to reviewer #3 (3.7), #4 (4.1) and #5 (5.4). It was necessary to develop a customized data charting form as the JBI templates were too rigid and it was necessary to tailor this form to our specific research question. We chose the variables in collaboration with our stakeholders including hospital administrators interested in implementing HaH solutions in the future. We will contact authors to request missing information if there is missing data. For situations whereby there are multiple publications for the same study these reports will be combined and described as the one HaH concept. This has been added to the text (line 125-126, page 7):

*“For situations whereby there are multiple publications for the same study these reports will be combined and described as the one HaH concept.”*

**6.17 The authors describe how the data will be presented but do not specify how it will be analyzed. Will content analysis be used? Will there be a specific analytical framework? Please consult the JBI Manual.**

Response: Thank you for this comment. Other reviewers have also identified this issue (see reviewer #2.1). We have addressed this point (line 159-175, page 9).

7 Reviewer #7

**Thank you for the opportunity to review this manuscript. I appreciate the effort the authors have put into developing this scoping review protocol. Below are my suggestions for improvement:**

**7.1. Title, Abstract, and Research Question: I recommend explicitly including the Scandinavian context in the title, abstract, and research question to ensure transparency regarding the focus of the scoping review (ScR) and its PCC framework.**

Response: Thankyou for this comment. It improves the manuscript considerably. Please see response to reviewer #3.4 and 3.5, reviewer #6.4 and 6.8 and reviewer #8.1. Please see page 1 (line 2), 2 and 4 (line 84).

**7.2. Grey Literature in the Search Strategy: On page 7, the search strategy section discusses the inclusion of grey literature. However, this is not mentioned in the abstract. I suggest incorporating this aspect in lines 147 and 148 to maintain consistency.**

Response: Thankyou for identifying this oversight. We have now adjusted the abstract to include information about grey literature searches (line 41, page 2).

**7.3. Justification for ScR Methodology: A more robust justification for selecting the ScR methodology would strengthen the Materials and Methods section. This justification could also serve as a foundation for explaining why quality assessment is not conducted for the included studies. I suggest adding this explanation at the end of the search strategy section, as readers unfamiliar with the ScR methodology might question the absence of a quality assessment.**

Response: Thankyou for this suggestion. We have included the following (line 128-131, page 8):

*“The scoping review methodology was chosen for this review because stakeholders wanted to map the existing literature published within Scandinavia and identify gaps in HaH research. A scoping review was also deemed relevant when the aim was to compare organisational models and provide a broader, more descriptive synthesis of the research landscape for acute HaH patients in Scandinavia.”*

**7.4. Discussion on Grey Literature Inclusion: The rationale for the limited inclusion of grey literature is not entirely clear in the discussion section. If a justification is necessary, I recommend clarifying this in line 216, particularly regarding the phrase "to assess the degree of research," which could be more precise. Additionally, if the objective is to present an overview of Scandinavian Hospital-at-Home (HaH) components (as stated in line 218), would it not be relevant to include what was excluded in lines 214 and 215? A clearer explanation of these decisions would enhance the discussion.**

Response: We would like to acknowledge that there are limitations to our planned review including the grey literature search. We have adjusted the manuscript now to expand upon “degree of research” and the limitation of using the Scandinavia block. See line 204-208, page 11:

*“Limitations to this review include a search strategy targeting Scandinavia alone. However, our stakeholders had limited interest in non-published studies and were concerned about the quality and relevance of non-peer-reviewed documents. Grey literature can result in an overwhelming volume of material and therefore ClinicaTrials.gov was chosen in relation to reporting of coming randomized control trials and websites were chosen to ensure that we could identify possible internal reports or pilot projects.”*

8 Reviewer #8

**The research topic is of practical significance and has potential value for improving the HaH service system and guiding clinical practice. However, there are also some deficiencies, which affect the depth, breadth, and rigor of the research and need to be further improved.**

**8.1. Duplication in background elaboration: In the introduction part, background information such as the pressure on the healthcare system and aging is repeatedly emphasized, resulting in redundant content. It can be streamlined and integrated to highlight the key points and avoid repetition.**

Response: Thank you for your valuable feedback regarding the background elaboration in the introduction. We agree that there is repetition of certain points. In response to your comment and other reviewers (see reviewer 3.4 and 6.4) we have adjusted the introduction considerably.

**8.2. Limited scope: The research only focuses on the Scandinavian region and excludes studies from other countries, which may lead to the omission of valuable experiences and viewpoints. Is it possible to expand the search scope, include internationally representative studies, compare the differences in the HaH models in different regions, and provide a more comprehensive reference for the Scandinavian region?**

Thankyou for this comment. Please see response to reviewer #3.6 and reviewer #5 and #6

**8.3. It is recommended to conduct stratified comparison: Considering that factors such as the severity of patients' conditions and underlying diseases may affect the research results, further stratified comparison can be carried out. For example, control groups can be set up by stratifying according to disease types (such as cardiovascular diseases, respiratory diseases, etc.) and the severity of the disease (mild, moderate, severe), ensuring that the research results are more targeted and reliable, and can accurately reflect the effectiveness of the HaH model under different circumstances.**

Response: Thank you for your recommendations regarding conducting a stratified comparison. We agree that stratifying control groups according to disease types (e.g., cardiovascular diseases, respiratory diseases) and the severity of the disease (mild, moderate, severe) could provide more targeted and reliable insights into the effectiveness of the HaH model. We will consider this approach depending on the review’s findings. If it proves to be applicable, we will certainly implement this stratified comparison to enhance the robustness of our findings.

**8.4. Insufficient categorization of intervention measures: The description of HaH intervention measures is broad and lacks detailed classification. It should be further refined, for example, classified according to disease types, intervention intensity, service content, etc., to facilitate in - depth analysis of the effectiveness of different intervention measures and their applicable populations.**

Response: Thank you for your feedback. In response to your comment, we have carefully revisited this question. The changes we have made according to your comments here include information that can be seen in Tables 3 and 4. The row headed “intervention” will describe the intervention in broad terms and the suggestions you have made are included in the other columns. For example disease type can be seen in Table 3 - reason for admission and service content is explained in more detail in the other columns in Table 4 such as “what are the roles of healthcare providers”. See line 155-158, page 9.
