# Supplementary material for: Mapping the organisational and interventional framework for patients admitted to Hospital-at-Home for acute illness in Scandinavia – a scoping review protocol
Source: PLoS One. 2025 Aug 4;20(8):e0328645. doi: 10.1371/journal.pone.0328645 (PMC12321056; doi:10.1371/journal.pone.0328645)
Supplement: Attachment — Submitted filename: Response_to_Reviewers_auresp_4.docx [file pone.0328645.s008.docx]

PONE-D-24-27648R3

Mapping the organisational and interventional framework for patients admitted to Hospital-at-Home for acute illness in Scandinavia – a scoping review protocol

PLOS ONE

*Response:*

*We sincerely thank you and the reviewers for the detailed and constructive feedback provided throughout this review process. We appreciate the considerable time and effort invested in the careful reading of our manuscript and the thoughtful comments offered across several rounds of revision. In response, we have made the suggested adjustments and have worked to improve the clarity, coherence, and overall quality of the manuscript accordingly.*

**Response to feedback from the editor:**

*Our abstract is adjusted as suggested removing subheading and adding keywords and ethical considerations. We have added a sentence in the method sections as you suggested in your response to the first round of review (line 94-98, page 5-6).*

*“The development of this review protocol was undertaken in collaboration with key stakeholders who expressed a specific interest in synthesising evidence related to Hospital at Home services within the Scandinavian context. In response to their input, the original search strategy was refined to ensure greater relevance to the geographical and contextual focus of the inquiry. The current strategy reflects this adjustment, incorporating terminology and sources tailored to the Scandinavian healthcare setting.”*

*This is the abstract that you suggested, similar to reviewer #3, #6 and #8´s comments (line 22-45, page 2):*

*"Hospitals in Scandinavia increasingly face an enormous pressure to manage acute emergencies in adults affected by multimorbid disabling conditions and therefore at risk of developing adverse hospitalisation outcomes such as nosocomial diseases. In this context, there is a growing political interest in the region to develop alternative models of acute emergency care such as the Hospital-at-Home (HaH), all the more so as adult patients themselves are showing a pronounced interest in HaH. We are therefore planning a scoping review to map the HaH components and interventions delivered to patients when managing acute emergencies in adults with that hospitalization model. We will search the databases MEDLINE, Embase and CENTRAL (Cochrane Central Register of Controlled trials) to include peer-reviewed articles of studies on adults admitted to HaH for acute emergency care within 24 hours of getting in touch with either an adult emergency department, an out-of-hours doctor, or a general practitioner. No limitation will be placed on the search period. The electronic search will be supplemented by a grey literature search of ClinicalTrials.gov and of the archives of Ministries of Health of the Scandinavian region. The information recorded during the data extraction process will include mainly the organisational structures of HaH, interventions delivered to adults with acute emergencies in HaH, and the characteristics of those patients. Data will be synthesized narratively. This protocol has been registered with Open Science Framework (DOI: 10.17605/OSF.IO/WKE7X). Mapping data on HaH for the care of adult acute emergencies in Scandinavia will help provide Scandinavian healthcare stakeholders with an overview of the strengths and weaknesses of different existing HaH models so that they can they can integrate that knowledge to make context-specific recommendations about and subsequently formally implement the management of adult acute emergencies in HaH in the region.*

*Ethical approval was not required as the study does not involve human participants. Findings will be submitted for publication in a peer-reviewed Scandinavian journal and disseminated through institutional websites and LinkedIn. Results will be presented at an international conference."*

**Reviewer #3**:

I would like to thank the authors for submitting a revised version of their manuscript. Below are my comments for further improvements:

1) When registering the study on OSF, the authors should complete the registration form directly on the platform rather than uploading a separate protocol document. For reference, see this example registration: <https://doi.org/10.17605/OSF.IO/RQKTP>

*Response: Thank you for your comment. We have now corrected this by completing the registration form directly on the OSF platform, as recommended.*

2) While reviewing abstracts of scoping reviews published in PLOS One, I noted that some use an unstructured format. The authors may consider reordering their abstract to begin with the introduction followed by the objectives to improve logical flow.

*Response: Thank you for your helpful comment. In line with the editor’s comment, we have revised the abstract to follow an unstructured format. We have used the version suggested by the editor. See line 22-45, page 2.*

3) The two review questions would benefit from being numbered (e.g., "1)", "2)") rather than presented in a single paragraph to enhance readability.

*Response: Thank you for your helpful suggestion. We have revised the manuscript by numbering the two review questions “1)” and “2)” instead of presenting them in a single paragraph, to enhance clarity and readability. See line 83-85, page 5.*

4) The exclusion of grey literature remains problematic. If stakeholder preferences preclude its inclusion, the authors should either:
a) Transition to a systematic review with a risk of bias assessment and GRADE analysis, or
b) Reframe the study as a narrative literature review.
Scoping reviews require exhaustive searches; limiting sources may compromise the methodology’s validity.

*Response: I think there has been a misunderstanding. We did not exclude grey literature. See line 34-35, page 2 and line 129-130, page 8.*

**Reviewer #5**:

Thank you for the updated and improved version of this manuscript. I have limited my comments to my own concerns rather than issues raised by other reviewers. I have answered "yes" to #3 and #4, above, but qualify this as "partly", as per my comments, below.

There is a mixture of future and past tense when describing the search strategy - “Will be reviewed” but then the paragraph notes that the the preliminary strategy was sent for review. The strategy seems to have been developed collaboratively rather than one information specialist responding to the PRESS checklist. There are processes to be followed when using PRESS (e.g., any required revisions necessitate a second formal review/PRESS). A reference to the PRESS checklist needs to be inserted and it would be helpful to include the completed PRESS so that it is clear how any issues were addressed.

I don’t think necessary to note that publications will be restricted to peer-review studies since this is implied through the choice of bibliographic databases.

*Response:* *We appreciate your comment regarding the phrase “restricted to peer-reviewed studies.” However, we respectfully maintain this wording to clarify our inclusion criteria from the databases (see line 123-124, page 8). In databases such as Embase, a substantial proportion of records consist of conference abstracts, preprints, and other forms of literature that have not undergone formal peer review. We hope we have clarified this now.*

The strategy provided actually uses MeSH headings rather than Emtree, so I suspect the preliminary strategy was developed in MEDLINE, not Embase, although this is not obvious without including the banner from the executed strategy. Only one of the headings uses an exclusively Emtree term, “Scandinavia”, as the MeSH is “Scandinavian and Nordic Countries” (Note: “Greenland” is not covered under this term and would require a separate entry in MEDLINE).

There are still deficiencies in the search strategy that should be addressed (several lines, e.g., 9, 10, 11, 19, 20, 21, 25, 32, 37, 41, 42, 46, should include both singular and plural options; some terms may appear without hyphens, e.g., homecare, homebased, hospitalbased, sameday; the regional terms should include Icelandic, Icelander*, Greenlander*, Nordic country; a mandatory wildcard should be used rather than an optional one for e.g., hospitali#ation.

*Response:*

*Thank you very much for your insightful comments regarding our search strategy and the distinctions between MeSH and Emtree terms in MEDLINE and EMBASE. After reflecting on your feedback, we carefully re-examined our search string.*

*You were correct that many of the MeSH terms originally included were specific to the MEDLINE database. We have now adjusted the search accordingly, including correcting wildcard use as suggested and broadening the country-specific block to ensure better coverage.*

*During this process, we discussed the difference between the .kf and .kw fields. Based on this, we have updated our search to use .kw to better reflect author-chosen keywords.*

*We also considered your helpful comments about wildcard use. Specifically, we explored whether the optional wildcard symbol (#) might help address variation in hyphenated terms such as home-based care. However, we found that the # symbol in Ovid substitutes only for a single optional character and is therefore not suitable for capturing common structural variations like home-based care, home based care, and homebased care. To ensure a more comprehensive and accurate search, we chose to explicitly include all three forms in our search string.*

*We sincerely thank you for the opportunity to revisit and improve our strategy. Your detailed and constructive feedback has significantly strengthened our search and contributed to our learning in the process. See Table 2, page 7-8.*

*Lastly, we have included a reference to the PRESS (Peer Review of Electronic Search Strategies) guideline to acknowledge best practice, although we have chosen not to include the full checklist, as its use is optional (line 112-113, page 7).*

*Thank you again for your valuable input.*

The methods also state the grey literature from ClinicalTrials.gov will be imported into Covidence – this is redundant with the study selection that follows so I suggest removal.

*Response: Although literature from Clinical Trials is grey literature, our stakeholders are specifically interested in any trials in the region that may be being conducted on this area.*

In discussing the limitations of the narrow grey literature, I would suggest using the term “missed” rather than “excluded”, which has a different meaning. Similarly, the note that the stakeholders had limited interest in non-peer reviewed documents is not connected to the restriction to the Scandinavian region, but rather to the grey literature. Note simply that the regional focus was considered central to the stakeholders’ interests, so was deemed necessary to e.g., avoid inflating volume. A targeted grey literature search was adopted for the same reasons.

*Response: Thanks for these comments. We have adjusted the manuscript accordingly - See line 127, page 8*

Some small grammatical notes:
Need hyphenation:
Table 1: out-of-hours doctor
Database-specific subject headings

*Response: Thanks. We appreciate you pointing out the grammatical issues. We have added the appropriate hyphenation in Table 1, correcting “out of hours doctor” to “out-of-hours doctor,” (line 104, page 6). “Database specific subject headings” has been deleted.*

Thank you for the opportunity to review this updated version.

**Reviewer #6:**

Dear authors,
I have received the revised version of your manuscript. Below are my observations.
First, I would like to take a moment to congratulate you on your work and to thank you for the effort dedicated to revising this protocol. Science is built through these spaces for discussion, and I appreciate your openness to critically engage with your own work.
Regarding the review of your manuscript, although most of the comments (both mine and those of the other reviewers) have been addressed, there are still some fundamental issues that should be revised before considering the work ready for dissemination. My final comments are as follows:

Abstract:
• If the abstract sections are determined by the journal’s structure, I suggest revisiting the content of each section. For example, in the methods section, it is not mentioned that this is a scoping review nor that it will follow the methodology proposed by JBI. This would be a good starting point. If there is no indication that a structured abstract is required, I suggest reorganizing the sections. Possible structure for consideration: Introduction (Objective), Methods, Expected Results (Implication of Results). However, you may reformulate it in the way that seems most appropriate, aiming to provide as much detail as possible within the word limit.

*Response:* *Thank you for your helpful suggestion. The abstract has been revised and reformulated to improve clarity and coherence, as also recommended by Editor and Reviewer #3 and #8. We have explicitly stated that the study is a scoping review and that it follows the methodology proposed by the Joanna Briggs Institute (JBI). These revisions have been made to ensure that the abstract provides a clear and structured overview of the study design and approach, in line with journal expectations. See line 27-28, page 2 and line 88-90, page 5.*

Introduction:
• I recommend providing more references to support the following statement: “Hospital-at-Home (HaH) has shown to reduce adverse events and distress 48 associated with hospitalization of adults and ease the burden on hospitals [4].” Additionally, the definition of HaH is only provided in the inclusion criteria section, which could result in readers unfamiliar with the term not understanding it upon first encounter. I recommend logically incorporating the definition of HaH within the introduction, and then referring back to it in the criteria section to avoid repetition (this strategy generally works well).
• Regarding the statement: “Healthcare systems are under pressure with an unprecedented number of people reaching retirement and living longer with multiple illnesses,” is this really the core problem? As phrased, it seems to suggest that living longer is the problem, when in reality, the issue is structural (as you note later). I suggest rethinking this idea.
• "One promising intervention is HaH." In line 54, this idea is introduced abruptly. Please review its integration into the text's logical flow.
• Line 61: A period is missing.
• Line 64: “Cochrane review.” Referring to the review this way may not be appropriate, as it appeals to the methodology rather than the study type, and creates an unnecessary distinction among reviews (not advisable). I suggest mentioning the authors or simply stating “a systematic review.”
• If there are no previous reviews in the Scandinavian context, it would be helpful to explicitly mention this. JBI recommends including the absence of prior reviews (reviews, not individual studies).

*Response:* *Thank you for your constructive and detailed feedback. We have carefully addressed all your suggestions:*

- *We have added additional references to support the statement regarding the benefits of Hospital-at-Home (HaH), thereby strengthening the evidence base (line 52-54, page 4).*
- *The definition of HaH has been added to the introduction for clarity and improved logical flow (line 55-58, page 4).*
- *The sentence about healthcare systems under pressure has been revised to clarify that the challenge lies in structural strain rather than longevity itself (line 52-55, page 4).*
- *The sentence “One promising intervention is HaH” has been integrated into the surrounding context, ensuring smoother narrative flow (line 51-52, page 4).*
- *The missing period has been inserted.*
- *We have replaced the term “Cochrane review” with “systematic review,” as advised, to avoid unnecessary methodological emphasis (line 66, page 4).*
- *Finally, we have explicitly noted the absence of previous reviews in the Scandinavian context, in accordance with JBI recommendations (line 72-73, page 4-5).*

General comment:
The logical and syntactic structure of the introduction remains unclear and may now be the main weakness of the manuscript. I suggest revising it. Avoid offering statements without proper contextualization. Short sentences can fragment the comprehension of meanings. Consider adopting a logical structure (deductive or inductive) and writing accordingly.

*Response: Thank you for your valuable feedback regarding the introduction. We have carefully revised this section to address your concerns. Specifically, we have improved the logical and syntactic structure by adopting a clearer, more deductive flow. We have also shortened the introduction to enhance readability and reduce fragmentation caused by overly short sentences. See line 49-81, page 4-5.*

Methods:
• Line 88: The "scoping review" methodology is not the JBI manual. The manual contains various methodologies. I suggest revising to: “methodology proposed by the Joanna Briggs Institute (JBI).”

*Thanks. See line 88-90, page 5*

• Line 91: “This protocol has been uploaded to Open Science Framework (DOI: 10.17605/OSF.IO/WKE7X).” Protocols are not “uploaded”; they are registered. This is correctly referenced in the abstract; please revise here.

*Thanks. See line 91, page 5*

Search Strategy:
• I was struck by the use of only “CENTRAL (Cochrane).” Will you not review systematic reviews? If not, it is not methodologically incorrect, but it should be clearly stated and justified. Additionally, I suggest providing the definition of CENTRAL (Cochrane Central Register of Controlled Trials).
• Line 112: “…controlled vocabulary thesaurus.” The expression "controlled vocabulary thesaurus" is redundant, as a thesaurus inherently represents a controlled vocabulary. It would be clearer to refer simply to "controlled vocabulary" or "thesaurus," depending on the intended emphasis.
• General comment: Review the “search strategy” section according to JBI methodology. This will help you report the procedures sequentially and logically.

*Response: We have received extensive feedback from Reviewer #3 and have revised the search strategy according to Reviewer #3 and JBI. Please see Table 2 for the altered search strategy*

• Line 114: Review the standardized use of acronyms (HaH).
• Line 116: If you have decided to include grey literature, it is inappropriate to state: “Publication type will be restricted to peer-reviewed.” I suggest revising this here and in other parts of the text where it appears.

*Response: Thank you for this comment. There has been some confusion about restrictions of literature type in this protocol article. This is a good opportunity to clarify it. Thank you. All non peer-reviewed publications such as conference abstracts and pre prints from Embase and MEDLINE were excluded. We chose a very selected type of grey literature (ClinicalTrials.gov and websites). We hope this is more clear in our manuscript. See the “Search strategy” section, line 106-132, page 6-9.*

• Line 118: “observational and descriptive.” Are there differences? What exactly do you mean by descriptive? This would also be a good point to clarify the comment regarding systematic reviews.

*Response: Thanks. We have clarified this. See line 125, page 8.*

• Lines 119–120: “Forward citation searches of included studies will be performed using the Web of Science database to identify any additional literature.” This statement is unclear. To which process are you specifically referring? Reverse citation searches? That is, searching references of potentially relevant articles? Or searching Web of Science more broadly? If it is the latter, WoS should be listed as a database in its own right (recommended). If not, please clarify.

*Response: Thankyou for the opportunity to clarify this. On discussion backwards citation searching should also be included. We have adjusted the manuscript accordingly and explained these two techniques (line 126-129, page 8). We are not using the WoS database more broadly and therefore has not included it in the list of databases in its own right.*

• Lines 121–124: “Grey literature from ClinicalTrials.gov will be imported into Covidence and screened with double-blinding. One author will screen any relevant website information and if considered relevant the information will be screened by a second author.” This paragraph should be included at the end of the “study selection” section.

*Response: Done. See line 145-146, page 9.*

• Lines 128–131: “The scoping review methodology was chosen for this review because stakeholders wanted to map the existing literature published within Scandinavia and identify gaps in HaH research. A scoping review was also deemed relevant when the aim was to compare organisational models and provide a broader, more descriptive synthesis of the research landscape for acute HaH patients in Scandinavia.” This paragraph should be removed from this section and logically integrated into the introduction as part of the review’s justification.

*Response: Done. Please see adjusted introduction. See line 49-85, page 4-5.*

• Line 140: Review according to the comment from “Line 88.”

*Response: Done. See line 88-90, page 5.*

• Lines 139–140: In the previous version, my comments suggested adding “agreement between reviewers during study selection.” Although the authors provided a response, it is not fully satisfactory according to the methodology. I will cite the section from the JBI manual I am referring to:
We recommend some pilot testing of source selectors prior to embarking on source selection across a team. This will allow the review group to refine their guidance or source selection tool (if one is being used). One framework for pilot testing is described below: - Random sample of 25 titles/abstracts is selected - The entire team screens these using the eligibility criteria and definitions/elaboration document - Team meets to discuss discrepancies and make modifications to the eligibility criteria and definitions/elaboration document - Team only starts screening when 75% (or greater) agreement is achieved.

*Response:* *Thank you for the helpful suggestion regarding the pilot testing process. We have intentionally kept the description somewhat broad to allow flexibility in the number of records screened during the pilot phase. This approach is intended to ensure that a sufficient number of disagreements can emerge and be discussed, thereby enhancing clarity and consistency in the application of the inclusion criteria before proceeding with full screening. We have added the following text to the manuscript (line 137-139, page 9):*

*“To support consistency in applying the inclusion criteria, a pilot process will be used that involve reviewers pausing to discuss an initial subset of records and uncertainties, and ensure screening alignment before proceeding with the full screening process”.*

Data Charting Process:
Now the process is clearer. As a final recommendation, I suggest adding that the variables to be extracted may be modified according to the findings. Remember that this is a protocol and it is always subject to change based on new insights.

*Response:* *Thank you for your helpful recommendation. We have now included a note in the manuscript stating that the variables to be extracted may be adjusted based on findings during the review process, in recognition of the fact that this is a protocol and subject to refinement as new insights emerge. See line 155-156, page 10.*

Data Analysis:
There are fragments that refer again to the data extraction process. Please review for clarity and avoid repetition.

*Response: Done. We have deleted the fragments you refer to.*

Discussion:
Ensure that the discussion does not simply extend the introduction. I suggest describing how the results will impact the various areas related to this investigation.

*Response: You are correct. We have deleted the first section (6 lines) and added description about how the results will impact the various areas. See line 185-191, page 11.*

*“Conducting an analysis that results in models describing the different ways HaH is organised across Scandinavian countries will provide valuable insights for stakeholders involved in planning and implementing such services. By identifying and comparing key components—such as staffing structures, referral pathways, clinical responsibilities, and integration with primary and specialist care—these models can highlight effective strategies as well as context-specific challenges. This knowledge can support evidence-informed decision-making, enable adaptation to local health system structures, and ultimately facilitate more efficient and acceptable implementation of HaH tailored to the Scandinavian context”.*

Line 184: Isn’t offered a definition to “EDs”

*Response: Corrected*
________________________________________
Final Comment:
The manuscript is now clearer. I encourage you to critically engage with the observations made, not treating them as rigid recommendations, but rather as perspectives that may strengthen your internal debate. Since this is a protocol and does not yet present results, you should take advantage of the opportunity to write with sufficient detail about all planned procedures.

**Reviewer #8:**

The article has undergone a remarkable revision, and I deeply value the authors' arduous efforts. Nevertheless, there remain several minor suggestions that require attention and resolution:

1. Abstract section: The content is relatively concise and covers the research purpose. However, it would be beneficial to appropriately add a brief description of the research methods and expected results, enabling readers to have a more comprehensive and rapid understanding of the core key points of the research.

*Response: Thank you. The abstract has been revised and reformulated to improve clarity and coherence, as also recommended by Editor, Reviewer #3 and #6. We have explicitly stated that the study is a scoping review and that it follows the methodology proposed by the Joanna Briggs Institute (JBI). These revisions have been made to ensure that the abstract provides a clear and structured overview of the methods. See line 22-45, page 2. We have not found it relevant to add something about expected results.*

2. Research selection: The process of literature screening using the Covidence platform is clear. However, there is no explanation regarding possible special situations during the screening process, such as how to handle documents in different languages and how to deal with the situation when the quality of the literature varies. Relevant content can be supplemented.

*Response: Thanks. This has been clarified. See line 122-132, page 8-9.*

3. Data analysis: The proposed data analysis method is relatively reasonable. However, when elaborating on how to organize the model according to data classification and iterative development, there is a lack of specific operation steps and examples. It can be further refined to make the research method more operational.

*Response: Thank you for your comment. We agree that the method for developing the models is uncertain at this stage, but this is inherent to the nature of the research. The approach will evolve iteratively based on the data we gather, and we will refine it as the analysis progresses.*
