# Supplementary material for: Mapping the organisational and interventional framework for patients admitted to Hospital-at-Home for acute illness in Scandinavia – a scoping review protocol
Source: PLoS One. 2025 Aug 4;20(8):e0328645. doi: 10.1371/journal.pone.0328645 (PMC12321056; doi:10.1371/journal.pone.0328645)
Supplement: Attachment — Submitted filename: Response_to_Reviewers_auresp_5.docx [file pone.0328645.s009.docx]

**Response to Reviewers**

*Dear Editor and Reviewer#5,*

*Thank you for the opportunity to further refine and improve our protocol. We truly appreciate the time and thoughtful feedback provided, which we believe will lead to a stronger and more rigorous final review*.

*We have carefully addressed the concerns raised in the latest round of review, as outlined below:*

Journal Requirements:

Please review your reference list to ensure that it is complete and correct. If you have cited papers that have been retracted, please include the rationale for doing so in the manuscript text, or remove these references and replace them with relevant current references. Any changes to the reference list should be mentioned in the rebuttal letter that accompanies your revised manuscript. If you need to cite a retracted article, indicate the article’s retracted status in the References list and also include a citation and full reference for the retraction notice.

Additional Editor Comments:

The authors have improved their manuscript and the reviewer has recommended acceptation of the manuscript for publication. However, there is a need for more precise organisation of the text, justifying the decision of minor revision before the manuscript can be accepted for publication.

1. Line 83: consider writing "Review questions" rather than "Review question".

*Response: Completed*

2. Inclusion and exclusion criteria sub-section of the Materials and Methods section: consider citing inclusion and exclusion criteria other than those derived from the PCC typology as well after table 2. Notably, the sentences "Any relevant studies...our review questions." (lines 122-126) and "Authors will be contacted...as the one HaH concept." (lines 130-132) should be inserted after table 2 given that they are highlighting inclusion and exclusion criteria (type of publication to be excluded, study designs to be included, and potentially exclusion of records for which full-texts will be unavailable even after contacting the author, at least for records retrieved from Medline and Embase). The authors should also state why they place a limitation on peer reviewed articles only for Medline and Embase databases, and not for the other electronic databases that will be searched. Even after reading the last sentence in lines 131 and 132, it is unclear to me how they will proceed for the inclusion of salami-sliced and duplicate articles. Consider also specifying that in the sub-section Inclusion and exclusion criteria. Information about the types of publications (editorials without data?) that will be excluded is also warranted.

*Response:*

*We understand that you would like all information regarding the inclusion and exclusion criteria to be consolidated in one location within the manuscript. In your comment, you referred to moving text "after Table 2," but we assume this may have been a typographical error and that you intended to refer to Table 1. In response, we have now summarized all relevant details about the inclusion and exclusion criteria in the paragraph accompanying Table 1. Our selection focused on original research identified through MEDLINE and Embase, to ensure that included studies provided sufficient detail—particularly on the organizational structures of HaH services. As a result, we excluded books, editorials, conference abstracts, duplicates and salami-sliced articles.*

*However, in recognition of stakeholder interest in a broader overview, we also included select relevant non–peer-reviewed sources. This rationale and the full criteria are now clearly presented on line 106-114, page 6-7.*

3. Data charting process sub-section
Consider subdividing the variables into three categories throughout the text and in the corresponding table: study characteristics, participant characteristics and main review outcomes, i.e., interventions and organisational structures of HaH. I suggest deleting tables 3 and 4 from the main manuscript given that they are just templates of the data extraction sheet. You could merge them in a single table titled "Template of data extraction sheet that will be used" in a way that information about the study characteristics is provided in arrows above, while the overview of interventions, organisational structures and patient outcomes are provided in the arrows below. You would then send that table to the appendix. Regarding the study characteristics to be mentioned in the template of data extraction sheet/preconceived data extraction sheet, consider adding the following variables: locality where the study was conducted (urban versus rural versus aemi-urban), sampling technique used, overall sample size and sample size of participants admitted to HaH, iinformation about the economic status of participants, and information about the comparator population of any.

*Response:*

*Thank you for your helpful and constructive suggestions regarding the “Data charting process” section. As requested, we have revised the manuscript to reflect your recommendations:*

- *Variables are now grouped into three categories:* ***study characteristics****,* ***participant characteristics****, and* ***main review outcomes*** *(interventions and organisational structures of HaH) and we have revised it consistently throughout the text. See line 36, page 2 and line 159, page 10*
- *Tables 3 and 4 have been removed from the main text. We have merged them into a single table titled “Template of data extraction sheet that will be used”, now placed in the S1 Table.*
- *Additional study characteristics have been added to the template (S1 Table), including: study locality (urban/semi-urban/rural), sampling technique, total and HaH sample sizes and comparator group details.*

4. Data analysis sub-section. It appears nowhere that you will conduct a narrative synthesis as expected in a scoping review protocol.

*Response: Thank you for your observation. We have now included on line 37, page 2 and line 165 and line 173, page 10 that the data will be synthesized narratively.*

5. As proposed in a previous round of revision, consider inserting a "Patient and public involvement" sub-section after the Data analysis sub-section, stating whether members of the public, people with lived disease experience of acute illness management in HaH. If you do not plan to include them, then simply write "None" under the "Patient and public involvement" sub-title. As highlighted in the previous round of revision, consider also inserting a "Protocol deviation"
sub-section just before the sub-section "Patient and public involvement" to specify how you would deal with any protocol modification after the publication of this manuscript. It is important to remind that protocol deviation is possible and this has been done since this protocol article has entered the review process. So, I find it appropriate to plan ahead.

*Response:*

*Thank you for the suggestion. We have revised the manuscript as requested:*

- *A “Protocol deviation” sub-section has been added before the Patient and public involvement section, outlining how potential modifications to the protocol will be addressed in future publications (line 180-183, page 11)*
- *A “Patient and public involvement” sub-section has also been inserted, stating that no patients or members of the public were involved in the development of this protocol (line 185-186, page 11)*

*We appreciate your guidance in improving the manuscript.*

6. Other comments
References.

I would delete reference 14 and use reference 23 for backing up the comment that was backed up by ref 14. Reference 24 is the same as reference 16.

*Response: Thank you. Reference 14 has been removed and replaced with reference 23. Reference 24 has been deleted.*

Line 149: consider replacing "using" by "by".

*Response: Completed*

Line 151: consider writing: "in a tabular form".

*Response: Completed*

Line 199: consider replacing "in the home" by "at home".

*Response: Completed*

Consider adding conflicts of interests and data availability statements before the reference section.

*Response: Thank you for the suggestion. We have now included the “Conflicts of interests” and “Data availability statement” sections prior to the reference section, as recommended. See line 239-244, page 13*

It is unclear to me whether you implemented Reviewer 3' s comment about the protocol registration on the OSF. I am unable to access the review protocol using the doi number provided in lines 38 and 92. Consider addressing this issue.

*Response: Thank you for drawing our attention to this. We have completed the registration of the review protocol on the Open Science Framework (OSF), as suggested by Reviewer #3. As you have pointed out the DOI number was incorrect. We have now corrected the DOI number accordingly in line 38, page 2; line 92, page 5 of the manuscript and in the PRISMA Checklist. The link is now accessible as intended.*

**Reviewer #5:**

Thank you for the opportunity to review the revised manuscript. I have limited my comments to my own concerns but note that other reviewers have generally responded positively to the most recent revisions which they requested. I have recommended "accept" but there are a few very minor adjustments that could be made pertaining to the search methods and strategy.

*Response:* *Thank you for your positive assessment and for recommending acceptance of the revised manuscript. We appreciate your thoughtful review and have carefully considered your remaining suggestions regarding the search methods and strategy. We have made the minor adjustments as recommended to ensure clarity and completeness.*

I disagree with the comment "We will search CENTRAL rather than the full Cochrane Library as our interest is limited to peer-reviewed original studies which CENTRAL specifically indexes". CENTRAL includes preprints and records from clinical trial registries, which would not be peer reviewed.

*Thank you for this helpful observation. We agree that CENTRAL includes records from clinical trial registries and may also index preprints or studies that have not yet been peer-reviewed. Our intention in selecting CENTRAL was based on its comprehensive indexing of randomized and controlled trials, which aligns with our focus on identifying original research.*

*However, we acknowledge that CENTRAL does not exclusively contain peer-reviewed studies. To address this, we have clarified our rationale in the manuscript to reflect that CENTRAL is being searched to identify original research studies, and that we will apply additional eligibility criteria during screening to exclude non–peer-reviewed records such as preprints.*

*We have updated the manuscript to reflect this more accurate reasoning in line 106-114, page 6-7.*

Within the search strategy, the following is noted:

exp Hospital to Home Transition/ (there are no subtrees so shouldn't explode - no effect on search)

By exploding "Scandinavian and Nordic Countries" - all but "Greenland" are already covered so listing them as separate MeSH is redundant. The only one required is "Greenland/"

Suggest adding truncation to pick up plurals for Norwegian*, Northern Europe*, and consider adding Swede, Swedes

*Response: Thank you for your helpful suggestions. We have revised the search strategy accordingly – see Table 2 in the “Search strategy” section, page 7-8.*
